# Kurarinone and sophoraflavanone G from *Sophora flavescens* suppress *Streptococcus agalactiae* biofilm via dual antibiofilm mechanisms involving adhesion protein binding and ABC transporter pathway perturbation

**DOI:** 10.3389/fmicb.2026.1905109

**Published:** 2026-08-31

**Authors:** Jihao Shi, Yun Zhang, Junxia An, Linglin Li, Anping Li

**Affiliations:** 1 College of Pharmacy and Food, Southwest Minzu University, Chengdu, China; 2 Academy of Agricultural and Forestry Sciences, Qinghai University, Xining, China

**Keywords:** ABC transporter, adhesion proteins, bovine mastitis, dual antibiofilm action, prenylated flavonoids, *Sophora flavescens*, *Streptococcus agalactiae*

## Abstract

**Introduction:**

Biofilm formation by *Streptococcus agalactiae* (*S. agalactiae*) is a major driver of chronic bovine mastitis that resists conventional antibiotic treatment, yet targeted strategies that disarm its biofilm machinery remain limited. *Sophora flavescens (S. flavescens)* is a medicinal plant rich in prenylated flavonoids with antimicrobial properties, but the antibiofilm mechanisms of these compounds against *S. agalactiae* remain unexplored.

**Methods:**

The antibacterial activity of 15 prenylated flavonoids from *S. flavescens* was evaluated against major mastitis-associated bacteria, and minimum inhibitory concentrations (MIC₉₀) were determined. Biofilm inhibition was assessed by crystal violet and acridine orange staining. Molecular docking, quantitative real-time PCR (qPCR), untargeted metabolomics, and phenotypic validation assays were integrated to elucidate the antibiofilm mechanisms. A mouse mastitis model was employed to evaluate *in vivo* efficacy, and cytotoxicity and acute toxicity assays were performed to assess safety.

**Results:**

Kurarinone and sophoraflavanone G exhibited potent antibacterial activity against *S. agalactiae*, *Streptococcus dysgalactiae*, and *Staphylococcus aureus*, with MIC₉₀ values of 3.12 μg/mL against *S. agalactiae*, superior to norfloxacin (6.25 μg/mL). Mechanistically, these compounds acted through two complementary pathways: (1) strong binding affinity to the adhesion-related proteins FbsA, FbsB, FbsC, and Lmb in molecular docking analysis, with transcriptional downregulation of their encoding genes confirmed by qPCR, and (2) perturbation of the ATP-binding cassette (ABC) transporter pathway, as suggested by untargeted metabolomics, leading to markedly reduced secretion of extracellular polysaccharides (by up to 60%), capsular polysaccharides, and extracellular DNA. Notably, this dual antibiofilm activity occurred at sub-MIC concentrations, indicating an antibiofilm mechanism that operates independently of direct bacterial killing and is distinct from the previously reported membrane-disruptive effects of these compounds. In a mouse mastitis model, intragastric administration of both compounds significantly ameliorated mammary tissue histopathology, reduced pro-inflammatory cytokines (TNF-*α*, IL-1β, IL-6, IL-8), and decreased myeloperoxidase activity, with favorable safety profiles (LD₅₀ > 2000 mg/kg).

**Conclusion:**

These findings suggest that adhesion proteins and the ABC transporter pathway represent potential dual targets against *S. agalactiae* biofilms, and position kurarinone and sophoraflavanone G as promising leads for antibiofilm drug development targeting biofilm-associated bovine mastitis.

## 1 Introduction

Bovine mastitis, a highly prevalent infectious disease in dairy herds, remains a major challenge to global milk production and dairy product safety (Soteriades et al., 2019; Martí-De Olives et al., 2020). The disease is primarily caused by a range of bacterial pathogens, among which *Streptococcus agalactiae (S. agalactiae)* is of particular concern due to its high transmissibility, ability to form biofilms, and persistent survival in dairy environments (Lakew et al., 2019; Pang et al., 2017). Crucially, biofilm formation is recognized as a key virulence factor that not only facilitates chronic and recurrent intramammary infections but also confers significant resistance to conventional antibiotic therapy. This makes biofilm-associated *S. agalactiae* mastitis exceptionally difficult to eradicate.

Antibiotics have long been the mainstay for managing bovine mastitis. However, their widespread and sometimes irrational use has accelerated the emergence of multidrug-resistant bacterial strains, diminished therapeutic efficacy, and raised concerns about antibiotic residues in milk and dairy products (Zhang et al., 2022; Lacroix et al., 2023). These residues can enter the human food chain, contributing to the spread of antimicrobial resistance (AMR) among human pathogens and undermining consumer confidence in dairy products (Bacanlı, 2024; Chen et al., 2023). Consequently, there is an urgent need to develop novel therapeutic agents that can not only kill bacteria but also effectively disarm them by neutralizing key virulence factors such as biofilm formation (Paramasivam et al., 2023).

Plant-derived natural products have gained increasing attention as potential antibiotic alternatives, owing to their low propensity for inducing bacterial resistance, favorable safety profiles, and minimal residual risks in food products (Chen et al., 2022). Among them, prenylated flavonoids—a class of secondary metabolites characterized by lipophilic side chains—have attracted considerable interest for their broad-spectrum antibacterial, anti-inflammatory, and antioxidant activities (Boozari et al., 2019; Lv et al., 2023; Weng et al., 2024; Long et al., 2022). However, while some prenylated flavonoids have been reported to act on bacterial cell membranes, their potential to specifically target the biofilm-associated virulence mechanisms in mastitis pathogens, particularly *S. agalactiae,* remains unknown. This gap is critical because an antibiofilm strategy could overcome the treatment bottlenecks that conventional antibiotics fail to address.

*Sophora flavescens (S. flavescens)* is a medicinal plant widely distributed across East Asia and known to produce a variety of prenylated flavonoids. In our previous studies, we isolated a series of prenylated flavonoids from the roots of *S. flavescens* and demonstrated their potent antimicrobial activities against plant pathogens (An et al., 2024; An et al., 2025). Specifically, sophoraflavanone G and kurarinone exhibited antifungal activity against *Botrytis cinerea (B. cinerea)* by disrupting cell wall and cell membrane integrity (An et al., 2024), and sophoraflavanone G suppressed *Xanthomonas oryzae* pv. *oryzae (Xoo)* through inhibition of biofilm formation, chemotaxis, and energy metabolism (An et al., 2025). These findings, particularly the observation that sophoraflavanone G inhibited biofilm in a plant pathogen, prompted a critical but unanswered question: Could the same prenylated flavonoids also target biofilms of *S. agalactiae,* a pathogen of major importance in dairy production? If so, what are the underlying molecular targets and pathways? Answering these questions would not only expand the application of these compounds but also potentially reveal novel antibiofilm mechanisms with therapeutic relevance.

The present study was designed to achieve three objectives. First, we aimed to expand the library of prenylated flavonoids from *S. flavescens* by isolating nine additional compounds to complement the six previously obtained (An et al., 2024; An et al., 2025), and systematically evaluating their antibacterial activity against four major mastitis-associated bacterial species: *S. agalactiae, Streptococcus dysgalactiae (S. dysgalactiae), Escherichia coli (E. coli),* and *Staphylococcus aureus (S. aureus).* Second, and most critically, we sought to elucidate the detailed molecular mechanisms by which the most active compounds inhibit *S. agalactiae* biofilm formation, with a focus on identifying their specific protein targets and metabolic pathways. Finally, we aimed to validate the *in vivo* efficacy and safety of these compounds using a mouse mastitis model. By demonstrating that these compounds can suppress adhesion-related proteins at the transcriptional level while interfering with the ABC transporter pathway—as suggested by molecular docking, qPCR, and metabolomic analyses—this work provides a scientific basis for developing novel antibiotic alternatives specifically designed to overcome biofilm-associated bovine mastitis.

## 2 Materials and methods

### 2.1 Materials

#### 2.1.1 Plant material

The roots of *S. flavescens* were purchased from the Anguo Medicinal Material Market in Hebei Province in 2023 and identified by Prof. Yuelin Zhao of the Key Laboratory of Biochemistry and Molecular Biology in Universities of Shandong Province, Weifang University. A voucher specimen (No. K2023S001) has been deposited at the College of Pharmacy and Food, Southwest Minzu University.

#### 2.1.2 Bacterial strains

*S. aureus* Newman and *E. coli* ATCC 25922 were provided by the Lanzhou Veterinary Research Institute, Chinese Academy of Agricultural Sciences, Lanzhou, China. *S. agalactiae* ATCC 27956 and *S. dysgalactiae* ATCC 27957 were purchased from Yutai Biotechnology Co., Ltd. (Xinyang, China).

#### 2.1.3 Animals

Kunming mice (body weight: 18–22 g) for the acute toxicity assay and female lactating mice (postpartum day 7) for the mastitis model were obtained from the Laboratory Animal Center of Lanzhou University. All animal procedures were approved by the Animal Ethics Committee of Lanzhou University (Approval No. D2024-947) and complied with the ARRIVE guidelines, EU Directive 2010/63/EU, and the NIH Guide for the Care and Use of Laboratory Animals.

#### 2.1.4 Chemicals and instruments

All chemical reagents and solvents were purchased from Shanghai Sun Chemical Technology Co., Ltd. Absorbance measurements were performed using a microplate reader (800TS, BioTek Instruments, Inc., Winooski, VT, USA). NMR spectra were recorded on a Bruker AM-400 spectrometer (Bruker Inc., Karlsruhe, Germany) with TMS as internal standard. Mass spectra were acquired on a Bruker APEX II 49e mass spectrometer (Bruker Daltonics Inc., Billerica, MA, USA) with an ESI source. HPLC analysis was performed on an Agilent 1,200 system equipped with an InertSustain C_18_ column (5 μm, 5 × 250 mm). Semipreparative LC was conducted on an NP7005C instrument (Jiangsu Hanbang Technology Co., Ltd., Lianyungang, China) with a YMC-Pack ODSA column (10 × 250 mm). Column chromatography was performed using silica gel (200–300 mesh, Qingdao Marine Chemical Factory, Qingdao, China), Sephadex LH-20 (GE Healthcare Bio-Sciences, Pittsburgh, PA, USA), and ODS reversed-phase silica gel (40–60 mesh, YMC Co., Ltd., Kyoto, Japan). Quantitative real-time polymerase chain reaction (qPCR) was performed using 2 × SG Fast qPCR Master Mix (Shenggong Bioengineering Co., Ltd., Shanghai, China). Transmission electron microscopy (TEM) observations were performed using a JEM-1010 TEM (NEC, Japan). Myeloperoxidase (MPO) activity was determined using a commercial MPO assay kit (Jianjian Bioengineering Institute, Nanjing, China).

### 2.2 Extraction and isolation

The isolation of total flavonoids (210 g) was continued following the extraction and isolation procedure previously reported for the isolation of six prenylated flavonoids (compounds ST1–ST4, ST9, and ST10; An et al., 2024; An et al., 2025). The total flavonoids were dissolved in chloroform/methanol (1:1, v/v) and subjected to Sephadex LH-20 gel column chromatography with the same solvent to obtain three subfractions (A1–A3).

Fraction A1 was dissolved in 75% aqueous methanol and separated by ODS column chromatography (40–60 mesh) with 75% aqueous methanol as the eluent to afford Fr1. Fr1 was further eluted with petroleum ether/acetone (5:1, v/v) on silica gel column chromatography (200–300 mesh) to obtain compound ST15 (30 mg). Fraction A2 was dissolved in 65% aqueous methanol and separated by ODS column chromatography to afford Fr2 and Fr3. These were further separated by silica gel column chromatography with gradient elution of petroleum ether/ethyl acetate (5:1, 3:1, 2:1, 1:1, v/v) to give subfractions Fr2-1, Fr2-2, Fr2-3, Fr2-4, Fr3-1, Fr3-2, and Fr3-3. Fr2-1 and Fr2-2 were purified by silica gel column chromatography with petroleum ether/acetone (3:1, 2:1, v/v) to obtain ST11 (6 mg) and ST13 (20 mg). Fr2-3 and Fr2-4 were fractionated by semi-preparative LC with methanol/water (85:1, 87:1, 90:1, 93:1, v/v) to afford ST5 (6 mg), ST6 (5 mg), ST8 (6 mg), and ST14 (5 mg). Fr3-1, Fr3-2, and Fr3-3 were subjected to silica gel column chromatography with dichloromethane/methanol (50:1, 30:1, 20:1, v/v) to obtain ST12 (30 mg). Fraction A3 was dissolved in 60% aqueous methanol to give Fr4, which was further purified by ODS column chromatography to yield ST7 (30 mg).

All purified compounds were dissolved in deuterated solvents and analyzed by NMR and MS. Their structures were identified by comparison with literature data.

### 2.3 Phenotypic activity assays

#### 2.3.1 *In vitro* antibacterial activity assay

The antibacterial activity of the compounds was determined using the microdilution method with slight modifications (Weng et al., 2024). Target compounds were dissolved in Brain-Heart Infusion (BHI) broth (HB8297-8, Qingdao Hope Bio-Technology Co., Ltd., Qingdao, China) containing 0.25% (v/v) dimethyl sulfoxide (DMSO). Then, 50 μL of compound-containing BHI was added to each well of a 96-well plate, followed by 50 μL of bacterial suspension (absorbance at 600 nm (OD_600_) = 0.1), achieving a final concentration of 100 μg/mL for each compound. After incubation at 37 °C for 12 h, OD_600_ was measured and the inhibition rate was calculated. For compounds with an inhibition rate ≥90%, the MIC_90_ was determined by two-fold serial dilution. MIC₉₀ was used instead of the conventional MIC₅₀ because it provides a more conservative and unambiguous baseline for defining sub-MIC concentrations in subsequent antibiofilm assays, and represents a more therapeutically relevant benchmark for near-complete bacterial growth inhibition. The inhibition rate was calculated as:

$\begin{array}{l} \text{Corrected absorbance}={\mathrm{OD}}_{600\;(\text{bacterial suspension})}\\ -{\mathrm{OD}}_{600(\text{bacterial}-\text{free suspension})}. \end{array}$

Inhibition rate=(C−T)/C×100%.

Where *C* is the corrected absorbance of bacterial growth in the control group and *T* is the corrected absorbance in the compound-treated group.

#### 2.3.2 Growth curve assay

The effect of ST3 and ST4 on the growth of *S. agalactiae* was evaluated as described previously (Weng et al., 2024). Briefly, *S. agalactiae* was cultured in BHI broth at 37 °C to logarithmic phase. The bacterial suspension was diluted to an initial OD₆₀₀ of approximately 0.03 and added to 96-well plates containing ST3 or ST4 at final concentrations of 0.78, 1.56, 3.12, and 6.25 μg/mL. The control group contained bacteria without compounds. The plates were incubated at 37 °C with shaking at 160 rpm, a speed selected to provide adequate aeration while minimizing shear stress that could interfere with subsequent biofilm formation assays. OD_600_ was measured every hour for 12 h, and growth curves were plotted.

#### 2.3.3 Time-killing curve assay

Time-killing curves were determined according to previous methods (Weng et al., 2024). Bacterial cultures with an initial OD₆₀₀ of approximately 0.03 were prepared in BHI broth containing ST3 or ST4 (0.78, 1.56, 3.12, and 6.25 μg/mL) and incubated at 37 °C with shaking at 160 rpm. At 0, 2, 4, 6, 8, 10, and 12 h, aliquots were taken, serially diluted in sterile PBS, and plated on BHI agar. Colonies were counted after 12 h of incubation.

#### 2.3.4 Biofilm inhibition assay

Biofilm formation was assessed by crystal violet staining and acridine orange staining (Miladi et al., 2016; Jha et al., 2020). *S. agalactiae* suspension (OD₆₀₀ = 0.1) was added to 96-well plates with ST3 or ST4 at final concentrations of 0.39, 0.78, and 1.56 μg/mL and incubated at 37 °C for 72 h. For crystal violet staining, biofilms were washed with PBS, stained with 0.1% crystal violet (CAS: 548–62-9, Shanghai Macklin Biochemical Co., Ltd., Shanghai, China) for 15 min, and dissolved in 95% ethanol. Absorbance was measured at 570 nm. For acridine orange staining, biofilms were stained with acridine orange (SL7651, Beijing Solarbio Science & Technology Co., Ltd., Beijing, China; 1 mg/mL) for 30 min and observed under an upright fluorescence microscope.

### 2.4 Mechanistic investigation of biofilm inhibition

#### 2.4.1 Homology modeling and molecular docking

The three-dimensional structures of ST3 and ST4 were retrieved from the PubChem database and energy-minimized. For Lmb, the crystal structure was downloaded from the RCSB Protein Data Bank (PDB ID: 3HJT). For FbsA, FbsB, and FbsC, no experimentally determined structures have been reported; therefore, their three-dimensional structures were generated using AlphaFold prediction and homology modeling. Subsequently, homology modeling was performed using MOE software, with protonation states and hydrogen bonding networks optimized at pH 7.0 and 300 K using the LigX module. The optimal model was selected based on the GB/VI score and refined using the AMBER10/EH force field. The stereochemical quality of the refined models was assessed by Ramachandran analysis. The proportions of residues in favored regions were >90% for FbsA and FbsC, and 87% for FbsB, indicating acceptable model quality for molecular docking purposes (Supplementary Figure 1). Molecular docking was conducted using AutoDockTools, and binding energies and interaction patterns were analyzed. The four adhesion-related proteins FbsA, FbsB, FbsC, and Lmb were selected for docking based on their established roles in *S. agalactiae* biofilm formation and host-cell adhesion.

#### 2.4.2 qPCR

*S. agalactiae* was treated with ST3 or ST4 (0.78 and 1.56 μg/mL) at 37 °C for 12 h. After treatment, bacterial cells were harvested by centrifugation at 8,000 rpm for 5 min. The collected pellet was mixed with 100 μL of lysozyme (400 μg/mL) to facilitate cell wall digestion, and total RNA was then extracted using a Column Bacterial Total RNA Extraction and Purification Kit (B518655, Sangon Biotech Co., Ltd., Shanghai, China) and reverse transcribed into cDNA using an AMV First Strand cDNA Synthesis Kit (B532445, Sangon Biotech Co., Ltd., Shanghai, China). qPCR was performed using 2 × SG Fast qPCR Master Mix on a real-time PCR system. The primer sequences for *FbsA, FbsB, FbsC,* and *Lmb* are listed in Supplementary Table 1. Gene expression levels were calculated using the 2^-ΔΔCT^ method (*ΔCT = CT_target gene_ − CT_GAPDH_*; *ΔΔCT = ΔCT_treatment_ − ΔCT_control_*; Rao et al., 2013).

#### 2.4.3 Extracellular polysaccharides and capsular polysaccharide analysis

EPS production was measured as previously described (Shang et al., 2020). *S. agalactiae* was treated with ST3 or ST4 (0.78 and 1.56 μg/mL) at 37 °C for 24 h. After centrifugation (2,000 rpm, 15 min), water-soluble and water-insoluble EPS were extracted and quantified using the anthrone method with glucose as standard.

Capsular polysaccharides were observed by TEM. Bacteria were treated with ST3 or ST4 (1.56 μg/mL) for 6 h. This time point was selected to allow sufficient exposure for observing compound-induced ultrastructural changes while minimizing interference from extensive cell lysis, based on the growth inhibition and bactericidal kinetics observed in this study. Cells were then fixed with 2.5% glutaraldehyde at 4 °C for 4 h, and post-fixed with 1% osmium tetroxide for 4 h. After dehydration through a graded ethanol series, samples were embedded in epoxy resin, ultrathin sectioned (50–70 nm), double-stained with uranyl acetate and lead citrate, and examined (Guo et al., 2025).

#### 2.4.4 Extracellular DNA assay

eDNA was measured as described with slight modifications (Rice et al., 2007). *S. agalactiae* cultures containing ST3 or ST4 (0.78 and 1.56 μg/mL) were mixed with 6% (w/v) milk solution in BHI medium and incubated in 12-well plates at 37 °C for 48 h. After washing with PBS, biofilms were treated with 0.5 mol/L EDTA at 4 °C for 1 h, followed by extraction with TEN buffer (50 mmol/L). eDNA was then precipitated with ethanol and quantified.

#### 2.4.5 Metabolomics analysis

Biofilm samples were collected as described in Section 2.3.4 (Biofilm Inhibition Assay) after treatment with ST3 or ST4 (0.78 and 1.56 μg/mL) for 72 h at 37 °C. Briefly, 100 mg of biofilm was mixed with 1 mL of extraction solution (water/acetonitrile/isopropanol, 1:1:1, v/v/v), subjected to low-temperature ultrasonic extraction at 4 °C for 30 min, and centrifuged (12,000 rpm, 4 °C, 10 min). The supernatant was stored at −20 °C for 1 h to precipitate proteins, followed by centrifugation under the same conditions. The resulting supernatant was dried under vacuum, reconstituted in 200 μL of 30% (v/v) acetonitrile, and centrifuged. The final supernatant was analyzed by LC–MS. The metabolomic profiling was performed with the assistance of Jiangsu Sanshu Biotechnology Co., Ltd. All subsequent data processing and bioinformatic analyses were independently conducted in our laboratory.

Data were processed using Orthogonal partial least squares discriminant analysis (OPLS-DA) models. Metabolites with Variable importance in projection (VIP) > 1 and *p* < 0.05 were considered differentially abundant. Pathway enrichment analysis was subsequently performed using the KEGG database to identify significantly affected metabolic pathways, with a particular focus on those associated with biofilm matrix biosynthesis, including the ABC transporter pathway.

### 2.5 Safety and *in vivo* efficacy evaluation

#### 2.5.1 Cytotoxicity and acute toxicity assays

Cytotoxicity against RAW264.7 macrophages and HIEC cells was evaluated using the CCK8 assay (Xu S. et al., 2025; Kwon et al., 2021). Cells were seeded in 96-well plates and treated with ST3 or ST4 (1.56–100 μg/mL) for 48 h. CCK8 reagent was then added, and absorbance at 450 nm (OD_450_) was measured.

Acute toxicity was assessed according to OECD Guideline 423 (OECD, 2002). Kunming mice (*n* = 6 per group) were intragastrically administered ST3 or ST4 at 2000 mg/kg (dissolved in 5% DMSO in vegetable oil). Mice were observed for 7 days for mortality and behavioral changes. Body weight was recorded daily.

#### 2.5.2 Mouse mastitis model and MPO assay

The mouse mastitis model was established as previously described (Zhang et al., 2019). Thirty lactating mice (postpartum day 7) were randomly assigned to four groups: blank control group (NC, *n* = 6), infected model group (MC, *n* = 6), positive control group (PC, norfloxacin, 100 mg/kg, *n* = 6), and treatment group (*n* = 12). The treatment group was further divided into two subgroups receiving ST3 (kurarinone, 150 mg/kg, *n* = 6) and ST4 (sophoraflavanone G, 150 mg/kg, *n* = 6), respectively. The mice were anesthetized, and 100 μL of *S. agalactiae* suspension (5 × 10^8^ CFU/mL) was injected into the fourth pair of mammary ducts in the MC, PC, and treatment groups. The NC group received no bacterial injection. Successful model establishment was confirmed by determining the bacterial load in mammary gland tissue and visual observation of mammary gland swelling at 72 h post-infection.

After 72 h, the mice were administered daily intragastric doses for 5 days: the NC and MC groups received sterile saline; the PC group received norfloxacin (100 mg/kg); the ST3 group received kurarinone (150 mg/kg); and the ST4 group received sophoraflavanone G (150 mg/kg). At the end of the treatment period, mice were euthanized by cervical dislocation under deep anesthesia with sodium pentobarbital (50 mg/kg, intraperitoneal injection). Mammary glands were collected and divided: one portion was used for bacterial load quantification, and the other was fixed in 4% paraformaldehyde, embedded in paraffin, sectioned, and stained with hematoxylin and eosin (H&E) for histopathological examination.

MPO activity in mammary gland homogenates was determined using a commercial MPO assay kit according to the manufacturer’s instructions (Zhang et al., 2019).

### 2.6 Statistical analysis

All experiments were performed in triplicate. Data are expressed as mean ± SD. One-way ANOVA followed by Duncan’s multiple range test was performed using SPSS 22.0 (IBM Corp., Armonk, NY, USA). *p* < 0.05 was considered statistically significant. Graphs were generated with GraphPad Prism 10 (GraphPad Software, San Diego, CA, USA).

## 3 Results

### 3.1 Structural elucidation

**Figure 1:**
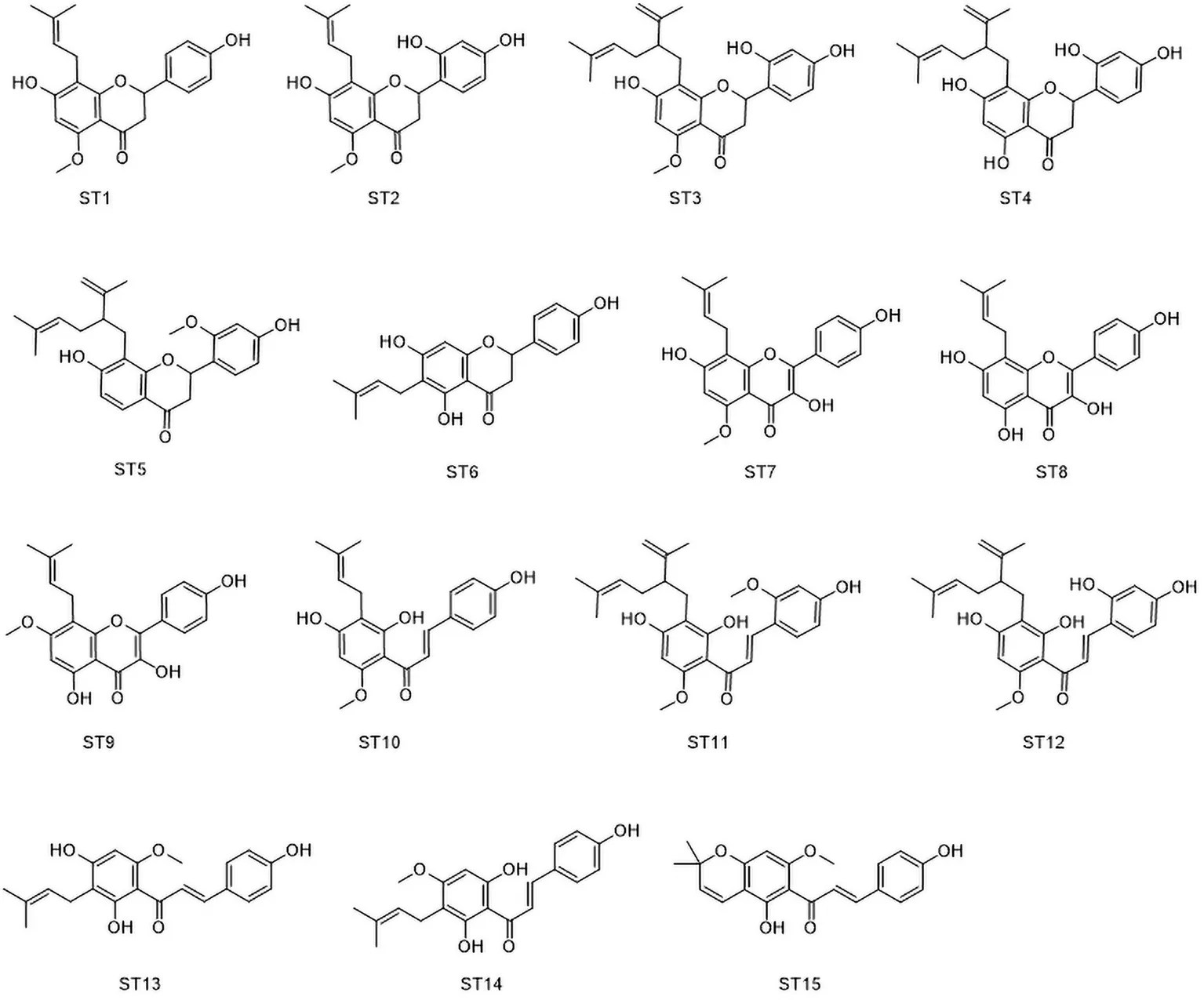
Chemical structures of compounds ST1–ST15 from *S. flavescens* investigated in this study.

In total, 15 known prenylated flavonoids from *S. flavescens* were investigated in this study (Figure 1). Six of these (ST1–ST4, ST9, and ST10) had been isolated and identified in our previous study (An et al., 2024; An et al., 2025). The remaining nine compounds, alopecurone G (ST5), 6-isopentenylnaringenin (ST6), sophoflavescenol (ST7), demethylanhydroicaritin (ST8), kushenol D (ST11), kuraridine (ST12), xanthohumol (ST13), xanthogalenol (ST14), and xanthohumol C (ST15)—are all known natural products previously reported in the literature, and were obtained from the isolation campaign conducted in this work. Their structures, along with those of the lead compounds ST3 and ST4, were confirmed by ^1^H NMR and ^13^C NMR analysis (Supplementary Figures 2–23) and HR-MS, with the detailed characterization data provided in the Supplementary data sheet and the corresponding literature references listed at the end of that file. The ^1^H and ^13^C NMR spectra of ST1, ST2, ST9, and ST10 have been previously published (An et al., 2024; An et al., 2025) and are not reproduced here. The absolute configurations of all compounds are as established in the cited literature.

### 3.2 Kurarinone and sophoraflavanone G exhibit potent antibacterial activity against mastitis pathogens

The antibacterial activity of all 15 compounds was evaluated against four major mastitis-associated bacteria (Supplementary Table 2). At 100 μg/mL, total flavonoids from *S. flavescens* completely inhibited the growth of *S. agalactiae* and *S. aureus.* Among the individual compounds, ST3 (kurarinone) and ST4 (sophoraflavanone G) fully inhibited the growth of *S. agalactiae, S. dysgalactiae,* and *S. aureus,* while ST2, ST10, and ST12 inhibited these three pathogens by over 90%.

The MIC₉₀ values of all test substances—including total flavonoids, compounds ST1–ST15, and norfloxacin—were determined by two-fold serial dilution against the four mastitis-associated bacterial species (Table 1). Among them, ST3 (kurarinone), ST4 (sophoraflavanone G), and ST12 exhibited the most potent activity against the three Gram-positive pathogens. For ST4, MIC₉₀ values ranged from 1.56 to 3.12 μg/mL across the three species. ST3 exhibited a consistent MIC₉₀ of 3.12 μg/mL against all three pathogens. ST12 showed MIC₉₀ values between 3.12 and 12.5 μg/mL. Notably, against *S. agalactiae*—the primary contagious mastitis pathogen—both ST3 and ST4 displayed MIC₉₀ values of 3.12 μg/mL, which was superior to that of the positive control norfloxacin (6.25 μg/mL). Based on this superior potency against *S. agalactiae*, together with their overall lower MIC₉₀ values compared with ST12, ST3 and ST4 were selected as lead compounds for all subsequent mechanistic and *in vivo* studies.

**Table 1:** MIC_90_ values (μg/mL) of total flavonoids, prenylated flavonoids from *S. flavescens*, and Norfloxacin against four mastitis-associated bacterial species.

Test substance	MIC_90_ value(μg/mL)			
	*S. agalactiae^a^*	*S. dysgalactiae^b^*	*E. coli^c^*	*S. aureus^d^*
Total flavonoids	25	25	>100	>100
ST1	25	25	>100	>100
ST2	50	50	>100	100
ST3	3.12	3.12	>100	3.12
ST4	3.12	3.12	>100	1.56
ST5	25	25	>100	25
ST6	25	25	>100	25
ST7	>100	>100	>100	>100
ST8	50	>100	>100	>100
ST9	>100	>100	>100	>100
ST10	25	25	>100	25
ST11	>100	>100	>100	>100
ST12	6.25	12.5	>100	3.12
ST13	25	>100	>100	>100
ST14	>100	>100	>100	>100
ST15	>100	>100	>100	>100
Norfloxacin	6.25	6.25	0.39	1.56

^a^*Streptococcus agalactiae* ATCC 27956; ^b^*Streptococcus dysgalactiae* ATCC 27957; ^c^*Escherichia coli* ATCC 25922; ^d^*Staphylococcus aureus* Newman.

### 3.3 Kurarinone and sophoraflavanone G Inhibit *S. agalactiae* growth and biofilm formation

#### 3.3.1 Growth inhibition and bactericidal kinetics

**Figure 2:**
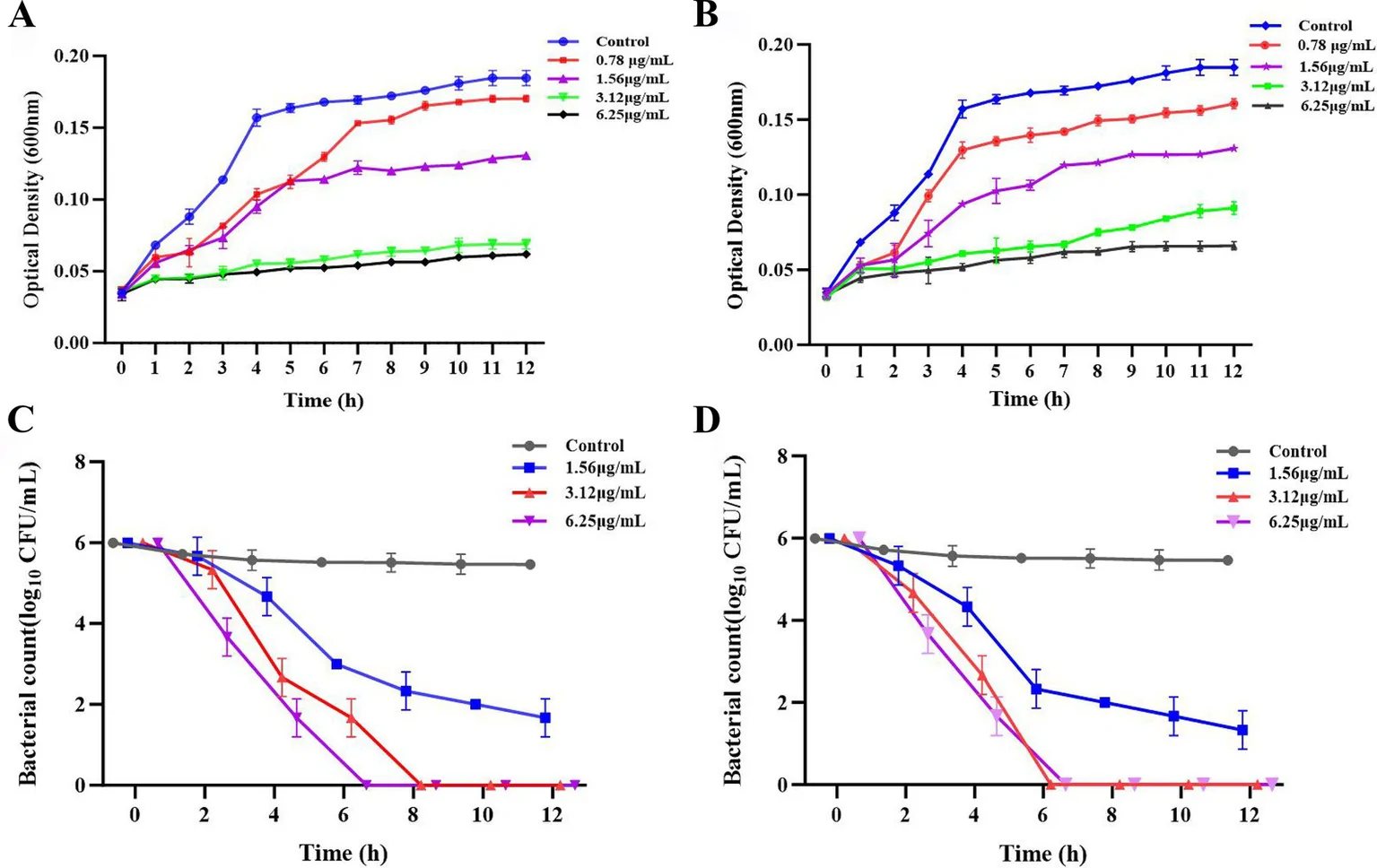
Effects of ST3 and ST4 on the growth of *S. agalactiae*. **(A,B)** Growth curves of *S. agalactiae* treated with ST3 **(A)** and ST4 **(B)** at 0.78–6.25 μg/mL; **(C**,**D)** Time-killing curves of ST3 **(C)** and ST4 **(D)** against *S. agalactiae*. Data are presented as mean ± SD (*n* = 3).

The effects of ST3 and ST4 on the growth of *S. agalactiae* were determined at different time points (Figure 2). Both compounds inhibited *S. agalactiae* in a dose-dependent manner. At 0.78 μg/mL, the logarithmic phase was shortened, but bacterial numbers in the stationary phase were similar to the control. At 1.56 μg/mL, the logarithmic phase was prolonged and the growth rate decreased. At 3.12 and 6.25 μg/mL, growth was almost completely inhibited.

The bactericidal kinetics of ST3 and ST4 against *S. agalactiae* were further investigated. At 1.56 μg/mL, the bactericidal activity and kinetic trends of ST3 and ST4 were largely consistent. At 3.12 and 6.25 μg/mL, ST3 achieved complete sterilization within 8 h and 6 h, respectively, whereas ST4 achieved complete sterilization within 6 h at both concentrations.

#### 3.3.2 Biofilm formation is suppressed at sub-inhibitory concentrations

**Figure 3:**
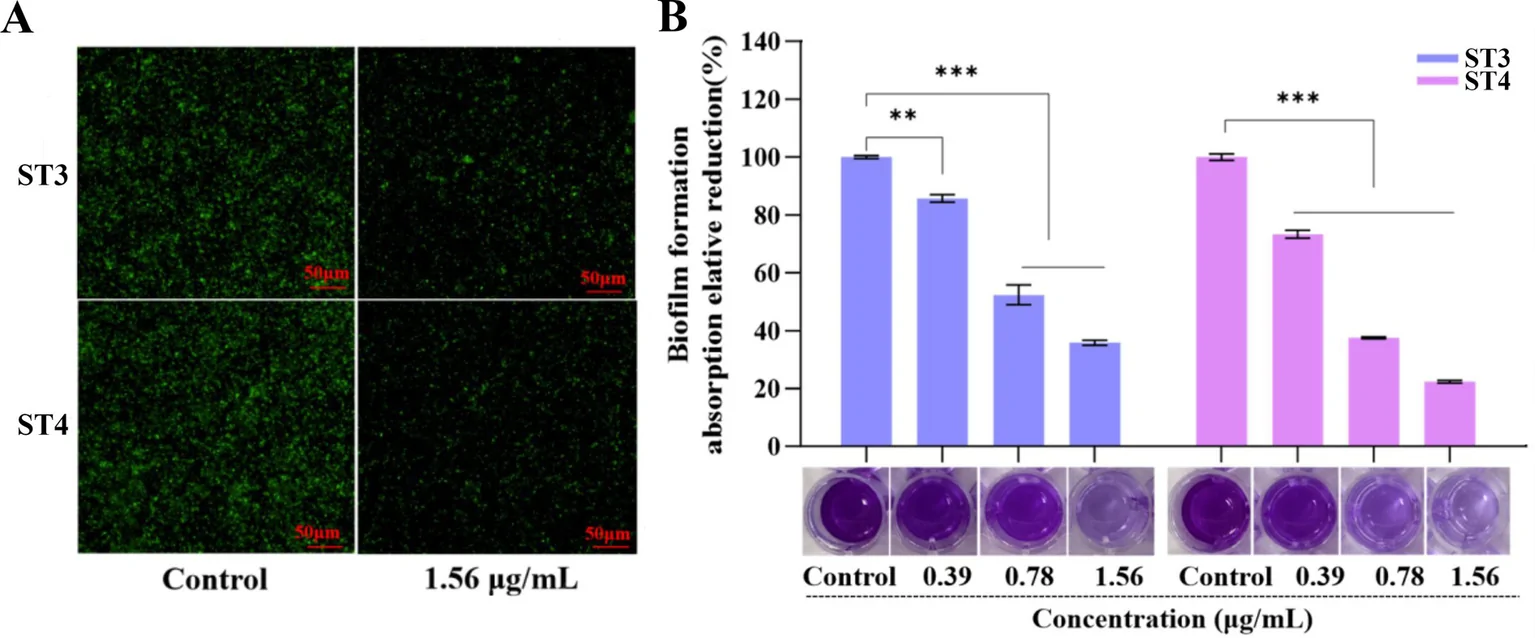
Effects of ST3 and ST4 on the biofilm of *S. agalactiae*. **(A)** Biofilm observed by acridine orange staining combined with fluorescence microscopy; **(B)** Biofilm quantification by crystal violet staining at 0.39–1.56 μg/mL. Data are presented as mean ± SD (*n* = 3). *p < 0.05, **p < 0.01, ***p < 0.001 vs. control.

To determine whether ST3 and ST4 could inhibit biofilm formation independently of their growth-inhibitory effects, biofilm assays were performed at sub-MIC concentrations (0.39–1.56 μg/mL). Acridine orange staining combined with fluorescence microscopy showed markedly reduced green fluorescence in both ST3- and ST4-treated groups compared with the untreated control, indicating decreased biofilm production (Figure 3A).

Crystal violet staining was used to quantify biofilm biomass. As shown in Figure 3B, biofilm staining intensity decreased concentration-dependently. For ST3, biofilm inhibition rates were 14.33, 47.66, and 64.18% at 0.39, 0.78, and 1.56 μg/mL, respectively. For ST4, the corresponding inhibition rates were 26.68, 62.53, and 77.64%, indicating consistently stronger antibiofilm activity than ST3 at all tested concentrations.

### 3.4 A dual antibiofilm action involving two complementary pathways

Having established the antibiofilm phenotype, we next sought to elucidate the underlying molecular mechanisms. Our investigations revealed two distinct but complementary modes of action.

#### 3.4.1 Pathway I: binding affinity to and downregulation of adhesion-related proteins

Given that bacterial adhesion is the initial and critical step in biofilm formation, we hypothesized that ST3 and ST4 might target adhesion-related proteins. Molecular docking was performed against four key *S. agalactiae* adhesins: FbsA, FbsB, FbsC, and Lmb.

Both compounds exhibited favorable binding energies to all four proteins, ranging from −6.3 to −8.9 kcal/mol (Table 2). The binding energies in descending order were FbsB (−8.9 kcal/mol for both) > FbsA (−7.6 for ST3; −7.7 for ST4) > Lmb (−7.3 for both) > FbsC (−6.3 for ST3; −7.1 for ST4). Notably, ST4 displayed considerably higher affinity for FbsC than ST3 (−7.1 vs. −6.3 kcal/mol), which may explain its stronger biofilm inhibitory effect (77.64% vs. 64.18% at 1.56 μg/mL). Detailed docking interactions, including hydrogen bonds and hydrophobic contacts with specific amino acid residues, are provided in Supplementary Figure 24.

**Table 2:** Binding energies (kcal/mol) of ST3 and ST4 to FbsA, FbsB, FbsC, and Lmb.

Compound	Binding energy (kcal/mol)			
	FbsA	FbsB	FbsC	Lmb
ST3	−7.6	−8.9	−6.3	−7.3
ST4	−7.7	−8.9	−7.1	−7.3

**Figure 4:**
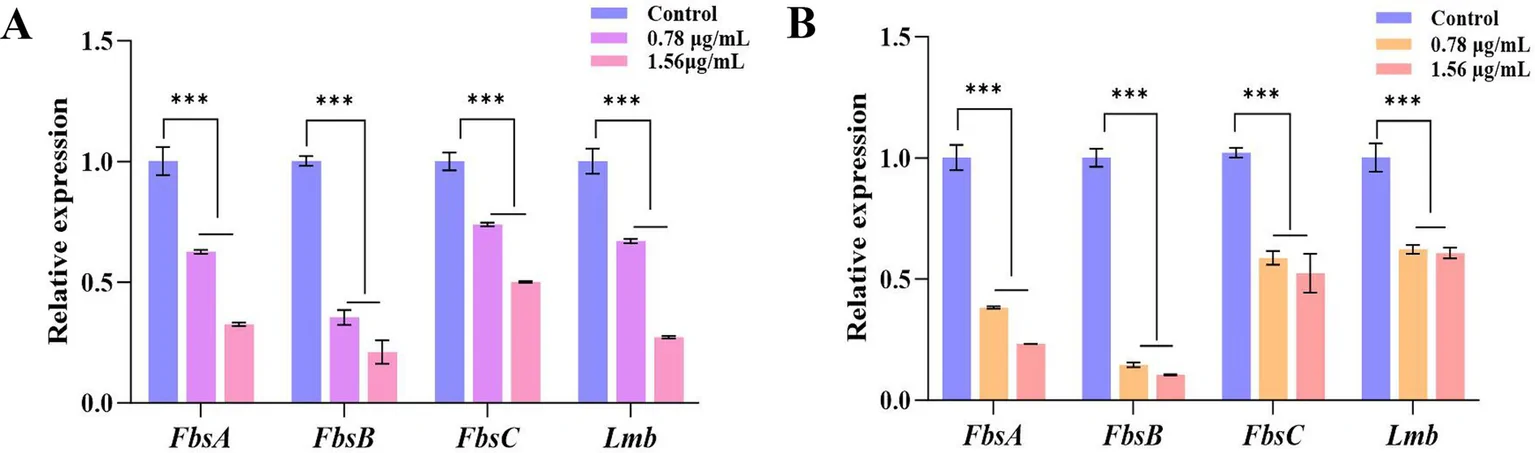
Effects of ST3 and ST4 on the mRNA expression of *FbsA, FbsB, FbsC,* and *Lmb* in *S. agalactiae*. **(A)** ST3 treatment at 0.78 and 1.56 μg/mL; **(B)** ST4 treatment at 0.78 and 1.56 μg/mL. Data are presented as mean ± SD (*n* = 3). *p < 0.05, **p < 0.01, ***p < 0.001 vs. control.

To validate these computational predictions, we examined the effects of ST3 and ST4 on the mRNA expression of *FbsA*, *FbsB*, *FbsC*, and *Lmb* by qPCR (Figure 4). Both compounds significantly downregulated all four genes in a dose-dependent manner, with the most pronounced effect on *FbsB*. At 0.78 μg/mL, ST3 reduced the expression of *FbsA*, *FbsB*, *FbsC*, and *Lmb* by 1.67-, 2.86-, 1.35-, and 1.49-fold, respectively, while ST4 decreased them by 2.63-, 6.67-, 1.70-, and 1.61-fold. At 1.56 μg/mL, ST3 further reduced expression by 3.03-, 4.76-, 2.00-, and 3.70-fold, and ST4 by 4.35-, 9.62-, 1.92-, and 1.64-fold. The greater potency of ST4, particularly against *FbsB* (9.62-fold at 1.56 μg/mL), is consistent with its superior biofilm inhibition activity.

#### 3.4.2 Pathway II: perturbation of the ABC transporter pathway and suppression of extracellular matrix components

**Figure 5:**
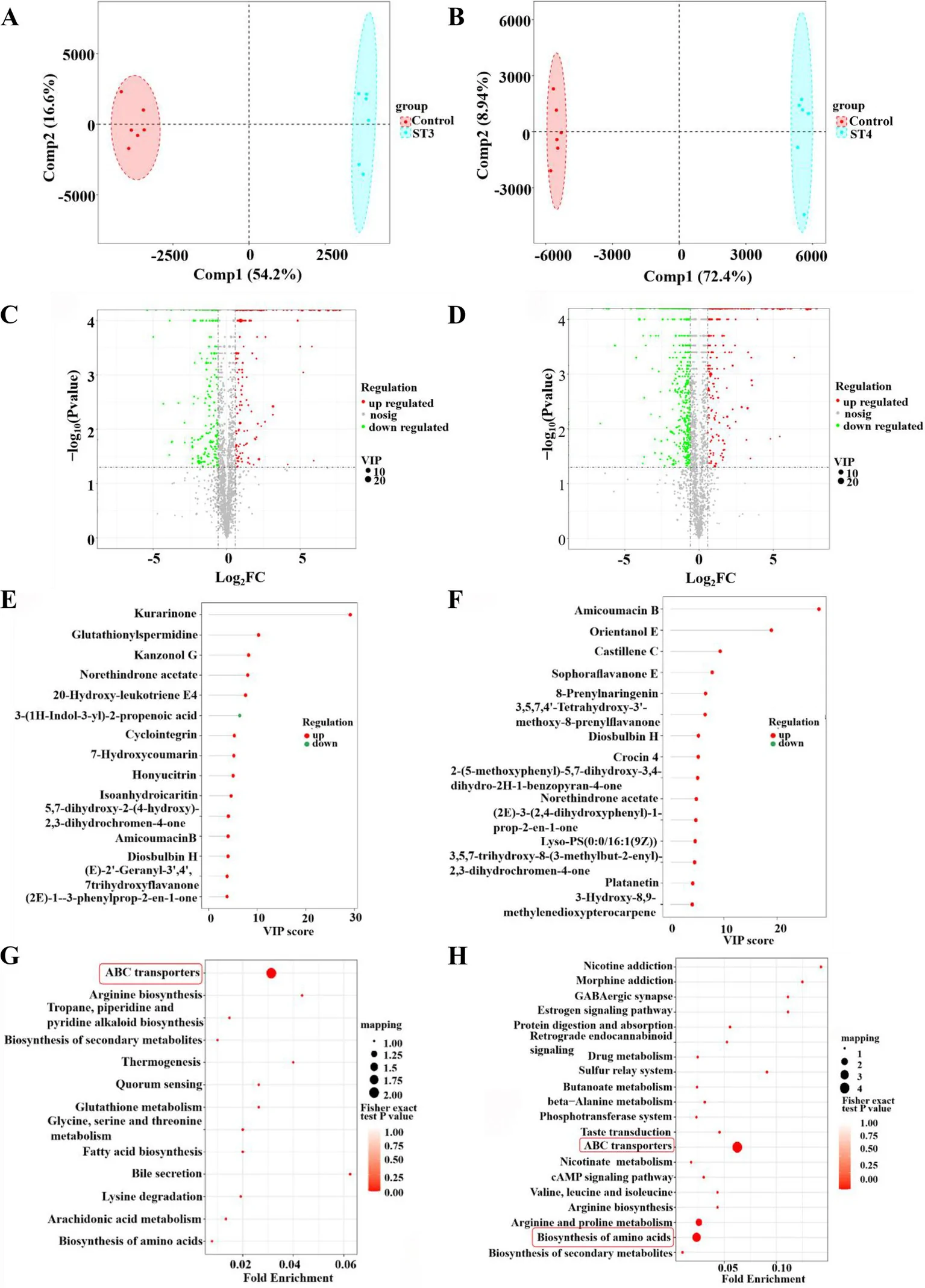
Untargeted metabolomic analysis of *S. agalactiae* biofilm treated with ST3 and ST4 at 0.78 and 1.56 μg/mL. **(A**,**B)** OPLS-DA score plots for ST3 **(A)** and ST4 **(B)** treatment groups versus the untreated control; **(C**,**D)** Volcano plots of differential metabolites in ST3 **(C)** and ST4 **(D)** groups; **(E**,**F)** Classification of differential metabolites in ST3 **(E)** and ST4 **(F)** groups; **(G**,**H)** KEGG pathway enrichment bubble plots for ST3 **(G)** and ST4 **(H)** groups. Metabolites with VIP > 1 and *P* < 0.05 were considered differentially abundant.

To identify additional pathways that might contribute to biofilm inhibition, we performed an untargeted metabolomic analysis of *S. agalactiae* biofilms treated with ST3 or ST4 (0.78 and 1.56 μg/mL). OPLS-DA score plots revealed clear separation between treatment groups and the untreated control, indicating substantial metabolic reprogramming (Figures 5A,B).

In the ST3-treated group, 499 significantly differential metabolites were identified (316 up-regulated, 183 down-regulated; Figure 5C), while in the ST4-treated group, 1,007 differential metabolites were identified (546 up-regulated, 461 down-regulated; Figure 5D). Among the differential metabolites, flavonoids accounted for 17.14% in the ST3 group (Figure 5E), including kurarinone, kanzonol G, and honyucitrin. Glutathionylspermidine and amicoumacin B were also identified among the differentially abundant metabolites. In the ST4 group (Figure 5F), sophoraflavanone E and 8-prenylnaringenin were enriched. The enrichment of flavonoids among the differential metabolites suggests that the administered compounds and their structural analogs may participate in the antibiofilm mechanism, as further discussed below.

Critically, KEGG pathway enrichment analysis identified the ABC transporter pathway as a significantly affected pathway in both treatment groups (Figures 5G,H). ABC transporters are known to be involved in the export of EPS—a major structural component of the biofilm matrix. This finding prompted us to investigate whether the perturbation of this pathway led to reduced EPS secretion and other matrix components.

**Figure 6:**
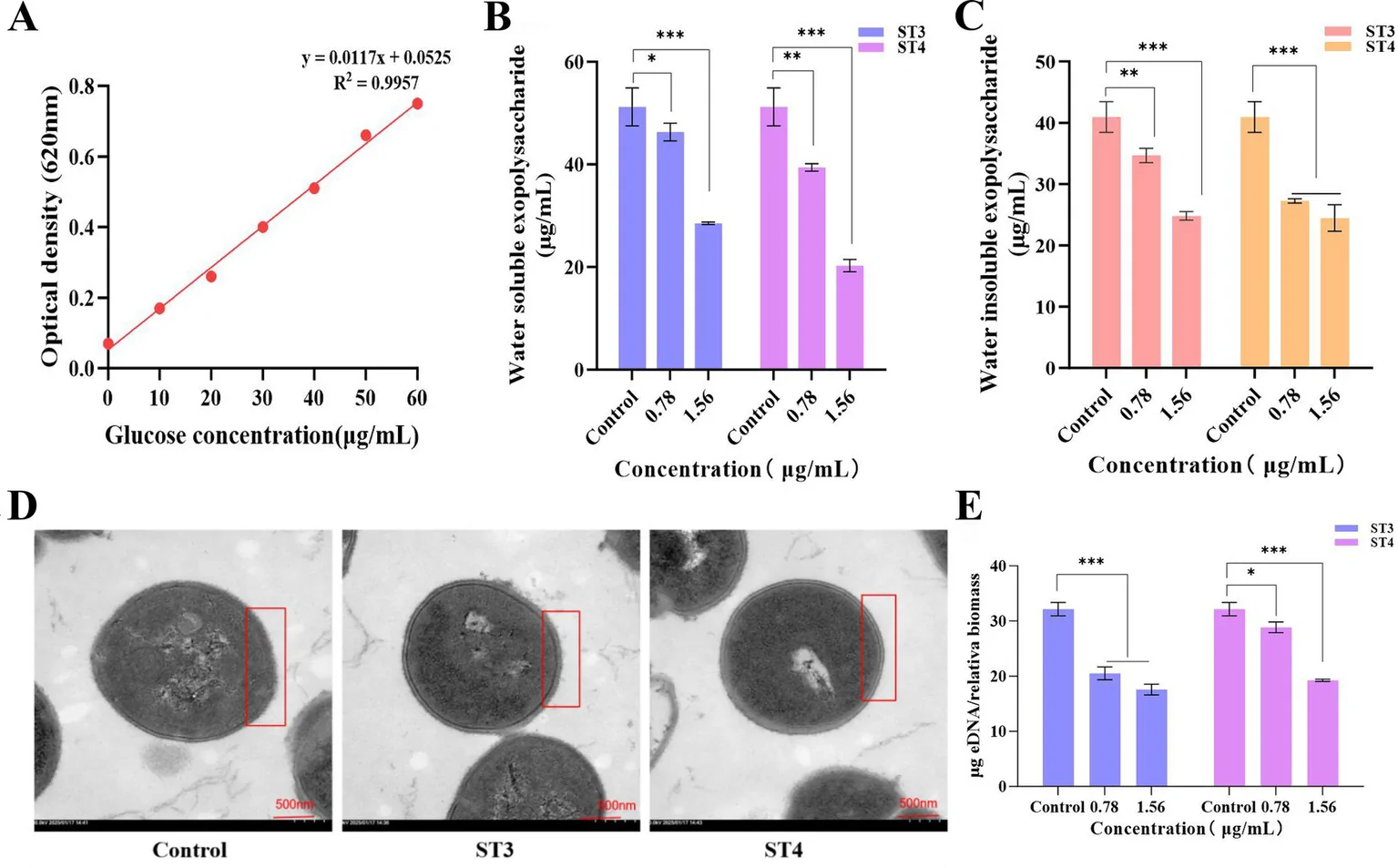
Effects of ST3 and ST4 on extracellular matrix components of *S. agalactiae* biofilm **(A)** Glucose standard curve for EPS quantification; **(B)** Water-soluble EPS levels at 0.78 and 1.56 μg/mL; **(C)** Water-insoluble EPS levels at 0.78 and 1.56 μg/mL; **(D)** TEM images of capsular polysaccharide morphology in untreated control, ST3-treated, and ST4-treated groups; **(E)** eDNA release at 0.78 and 1.56 μg/mL. Data are presented as mean ± SD (*n* = 3). *p < 0.05, **p < 0.01, ***p < 0.001 vs. control.

We first quantified EPS production. A glucose standard curve was constructed for EPS quantification (Figure 6A, *Y* = 0.0017*x* + 0.00525, *R^2^* = 0.9957). Both ST3 and ST4 significantly reduced water-soluble and water-insoluble EPS levels (Figures 6B,C). At 0.78 and 1.56 μg/mL, ST3 reduced water-soluble EPS levels by 9.61 and 44.30%, respectively, while ST4 reduced them by 23.06 and 60.43%. Similarly, for water-insoluble EPS, ST3 reduced levels by 15.34 and 39.40%, and ST4 by 16.68 and 40.22%.

TEM observations confirmed the impact on capsular polysaccharides, which are structurally related to EPS. In the untreated control group, the surface of *S. agalactiae* was densely covered with a thick capsular layer. After ST3 treatment, this layer was visibly reduced, and after ST4 treatment, the bacterial surface appeared nearly smooth, with almost complete loss of the capsular polysaccharide coat (Figure 6D).

Furthermore, both compounds reduced the release of eDNA, another critical biofilm matrix component. At 0.78 μg/mL, ST3 and ST4 reduced eDNA levels by 36.19 and 10.13%, respectively. At 1.56 μg/mL, ST3 and ST4 reduced eDNA levels by 40.32 and 40.14%, respectively (Figure 6E).

Taken together, these data suggest that ST3 and ST4 perturb the ABC transporter pathway, leading to a coordinated suppression of multiple biofilm matrix components—EPS, capsular polysaccharides, and eDNA—thereby compromising the structural integrity of the *S. agalactiae* biofilm.

### 3.5 Safety profile: low cytotoxicity and acute toxicity

**Figure 7:**
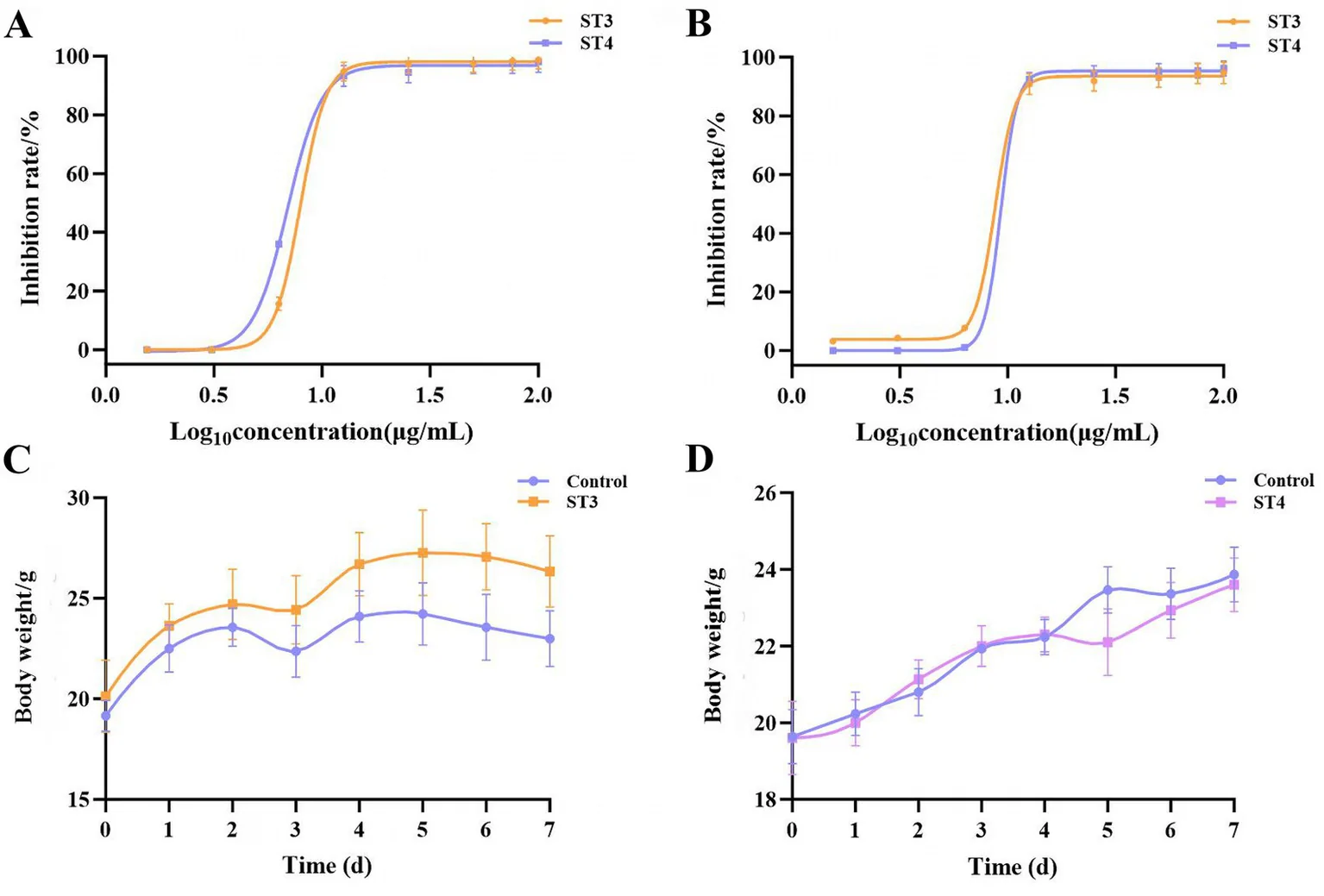
Cytotoxicity and acute toxicity evaluation of ST3 and ST4. **(A,B)** Cytotoxicity against RAW264.7 macrophages **(A)** and HIEC intestinal epithelial cells **(B)** assessed by CCK8 assay at 1.56–100 μg/mL; **(C,D)** Body weight changes in mice after a single intragastric administration of ST3 **(C)** or ST4 **(D)** at 2000 mg/kg compared with the control group. Data in **(A,B)** are presented as mean ± SD (*n* = 3). Data in **(C,D)** are presented as mean ± SD (*n* = 6 per group).

The safety of ST3 and ST4 was evaluated *in vitro* and *in vivo*. The effects of ST3 and ST4 on RAW264.7 macrophages and HIEC intestinal epithelial cells were assessed using the CCK8 assay (Figures 7A,B). At high concentrations (12.5 to 100 μg/mL), both compounds inhibited cell viability by more than 90%. However, at concentrations closer to their antibacterial effective range, cytotoxicity was substantially lower. At 6.25 μg/mL, inhibition rates for RAW264.7 cells were 15.57% (ST3) and 35.99% (ST4), and for HIEC cells were 7.71% (ST3) and 1.11% (ST4). At 1.56 and 3.12 μg/mL—concentrations directly corresponding to their MIC₉₀ values against *S. agalactiae*—no cytotoxicity was observed against RAW264.7 cells, while HIEC cells showed only marginal inhibition rates of 4.33% (ST3) and 3.27% (ST4), respectively. These data demonstrate a favorable selective toxicity profile, with a clear therapeutic window between antibacterial efficacy and mammalian cell cytotoxicity.

In the acute toxicity assay conducted according to OECD Guideline 423 (OECD, 2002), mice treated with a single intragastric dose of ST3 or ST4 at 2,000 mg/kg exhibited no mortality or abnormal behavior over a 7-day observation period. Body weight gain was comparable to that of the control group (Figures 7C,D). Both compounds therefore exhibited a median lethal dose (LD₅₀) > 2000 mg/kg, classifying them as low-toxicity agents.

### 3.6 Kurarinone and sophoraflavanone G ameliorate mastitis in a mouse model

#### 3.6.1 Histopathological improvements

**Figure 8:**
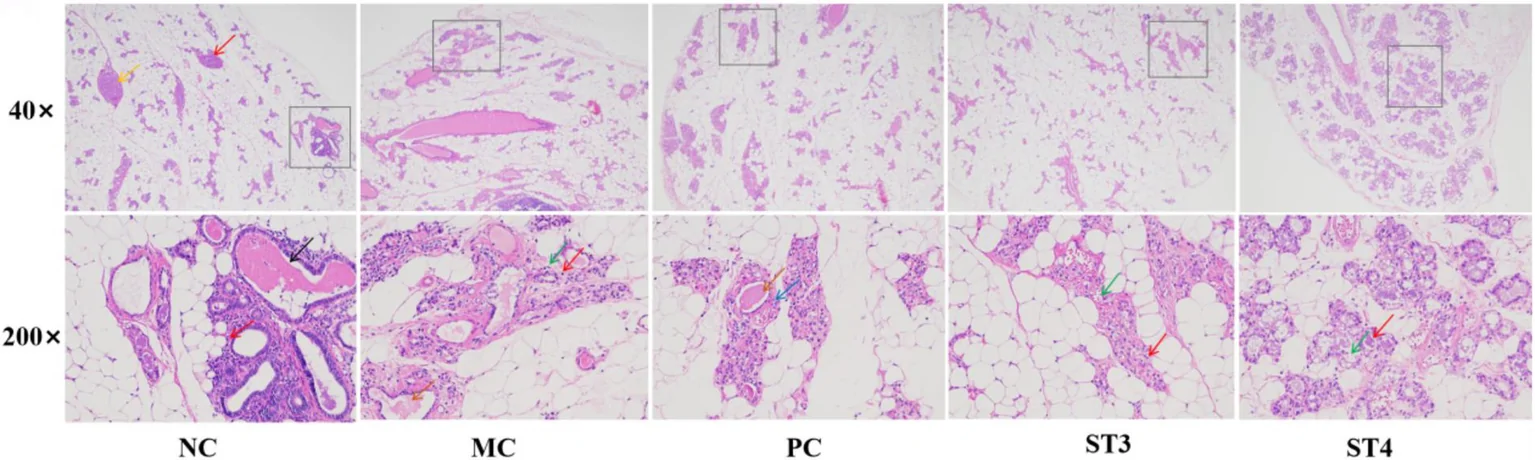
Histopathological changes of mammary gland tissues in ST3- and ST4-treated mice. NC: Blank control (uninfected); MC: Infected model; PC: Positive control (norfloxacin, 100 mg/kg); ST3/ST4: Treatment groups (150 mg/kg). Yellow arrows: Mild ductal dilation; brown arrows: Eosinophilic material deposition; black arrows: Necrotic cellular debris; red arrows: Lymphocytic infiltration; green arrows: Cytoplasmic vacuoles in epithelial cells. Black boxes in 40 × images indicate the locations of the 200 × magnification fields.

Histopathological examination of mammary tissues was performed by H&E staining (Figure 8). Compared with the uninfected blank control group, the *S. agalactiae*-infected model group exhibited marked pathological changes: increased numbers of acini with irregular arrangement, mild ductal dilation (yellow arrows), eosinophilic material deposition (brown arrows), necrotic cellular debris within acini and ducts (black arrows), and scattered lymphocytic infiltration in the stroma (red arrows). In contrast, the ST3- and ST4-treated groups showed substantially reduced histological lesions. Acinar counts were lower, many epithelial cells contained round cytoplasmic vacuoles (green arrows), and only minimal eosinophilic deposits (brown arrows) were observed in a small number of acinar lumens.

#### 3.6.2 Downregulation of pro-inflammatory cytokines

**Figure 9:**
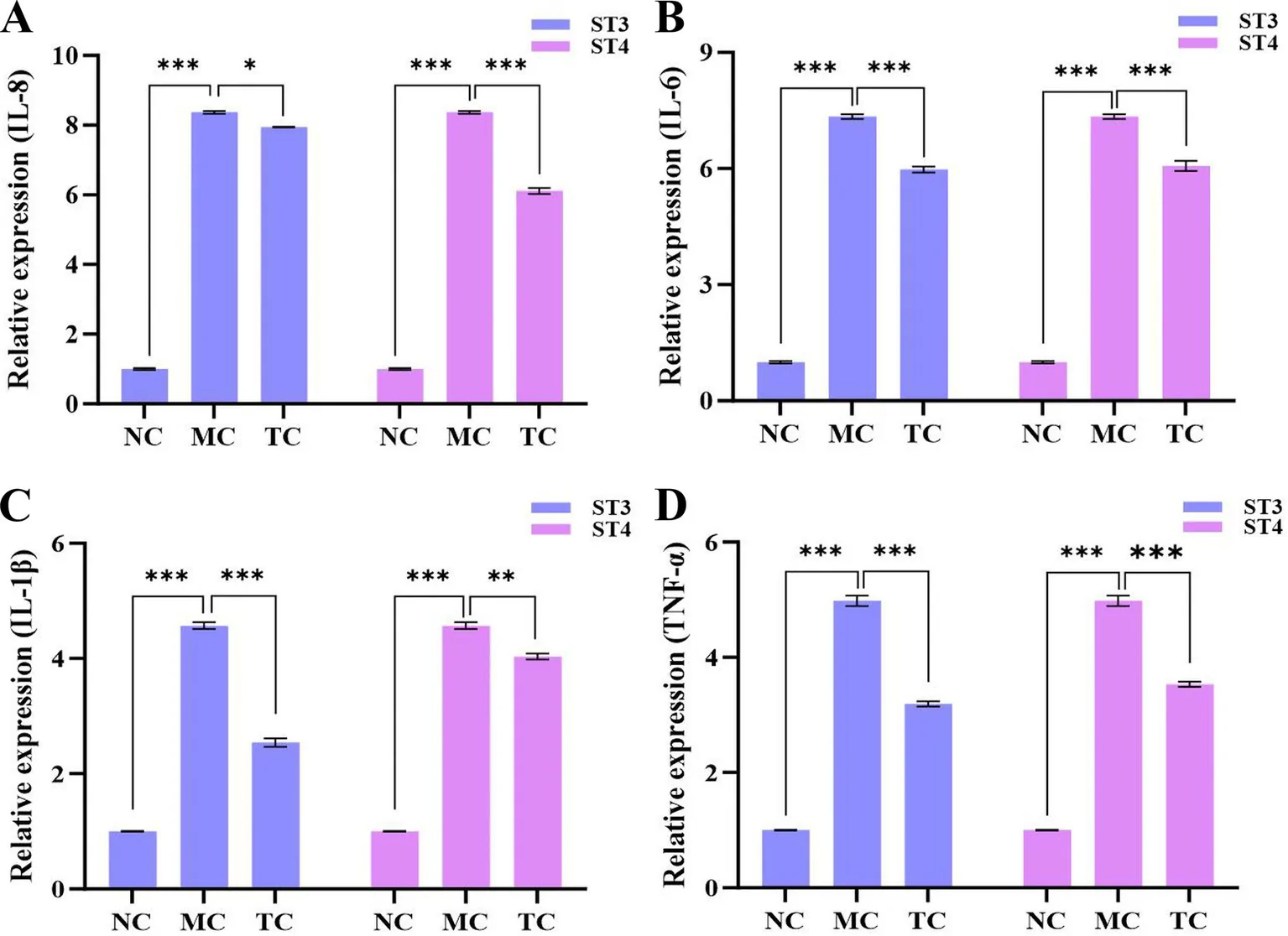
Effects of ST3 and ST4 on pro-inflammatory cytokine expression in mammary tissues of *S. agalactiae*-infected mice. **(A)** IL-8; **(B)** IL-6; **(C)** IL-1β; **(D)** TNF-*α*. NC: Blank control (uninfected); MC: Infected model; ST3: Kurarinone treatment (150 mg/kg); ST4: Sophoraflavanone G treatment (150 mg/kg). Data are presented as mean ± SD (*n* = 6 per group). *p < 0.05, **p < 0.01, ***p < 0.001 vs. NC/MC group.

Infection with *S. agalactiae* significantly upregulated the expression of pro-inflammatory cytokines in mammary tissue. As shown in Figure 9, treatment with ST3 or ST4 markedly attenuated this inflammatory response. Compared with the infected model group, ST3 reduced the relative expression of IL-6, TNF-*α*, and IL-1β by 19.05, 35.94, and 44.78%, respectively. ST4 reduced these cytokines by 17.41, 28.51, and 12.29%, respectively, and additionally reduced IL-8 expression.

#### 3.6.3 Reduction of MPO activity

**Figure 10:**
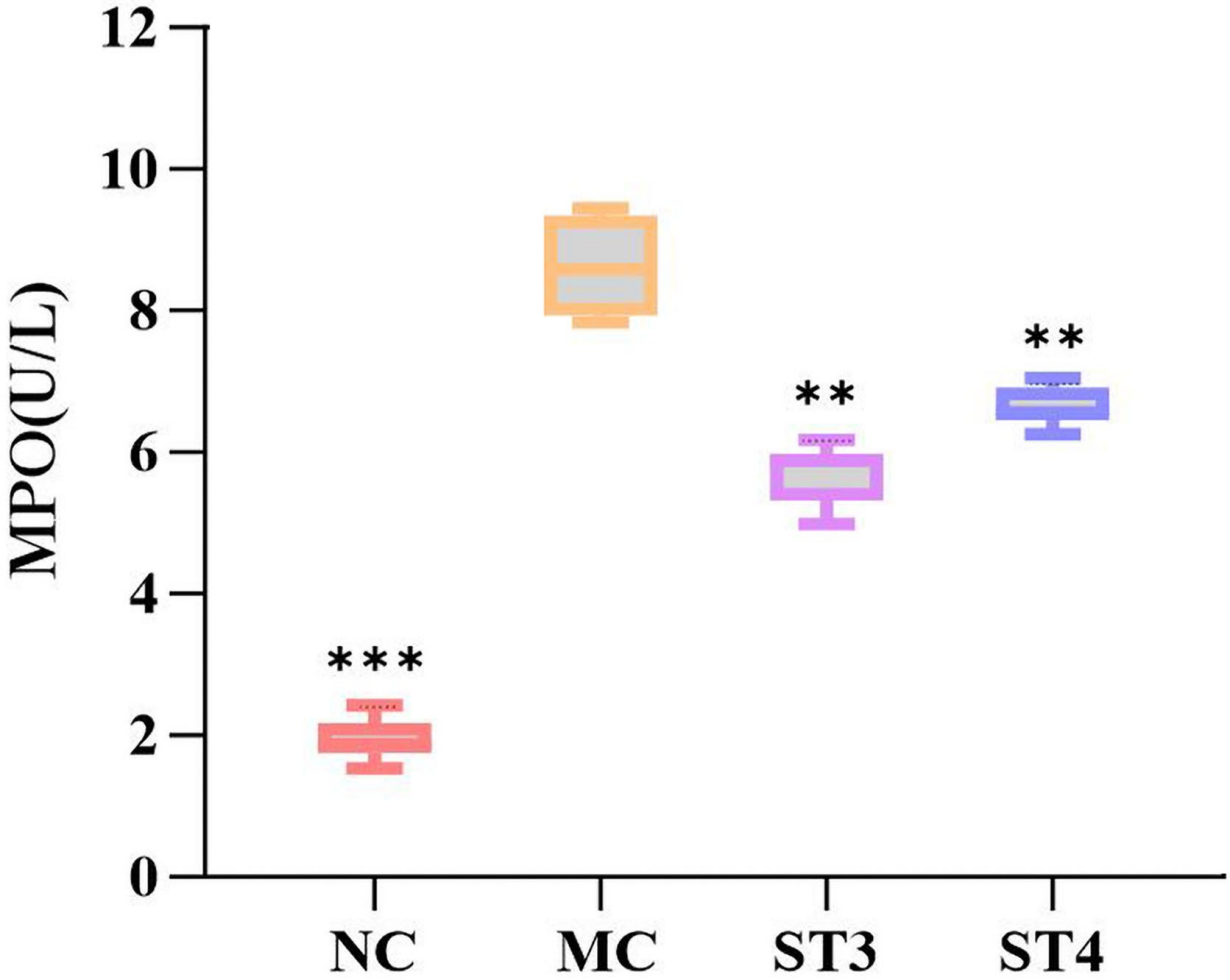
Effects of ST3 and ST4 on myeloperoxidase (MPO) activity in a mouse mastitis model. NC: Blank control (uninfected); MC: Infected model; ST3: Kurarinone treatment (150 mg/kg); ST4: Sophoraflavanone G treatment (150 mg/kg). Data are presented as mean ± SD (*n* = 6 per group). **p < 0.01 and ***p < 0.001 vs. MC group.

MPO activity, an indicator of neutrophil infiltration and inflammation, was significantly elevated in the infected model group compared with the blank control (uninfected). Treatment with ST3 and ST4 reduced MPO activity by 33.68 and 22.96%, respectively, compared with the infected model group, indicating reduced neutrophil-mediated tissue damage (Figure 10).

## 4 Discussion

In this study, we expanded our library of prenylated flavonoids from *S. flavescens* to a total of 15 compounds—including nine obtained from the isolation campaign conducted in this work—and identified kurarinone (ST3) and sophoraflavanone G (ST4) as potent antibacterial agents against major bovine mastitis pathogens. The most significant finding of this work is the demonstration of a dual antibiofilm action against *S. agalactiae*: binding affinity to and downregulation of adhesion-related proteins (FbsA, FbsB, FbsC, and Lmb) demonstrated through molecular docking and qPCR analysis, combined with metabolomic profiling and phenotypic validation of ABC transporter pathway perturbation leading to suppressed secretion of EPS and other biofilm matrix components. These findings suggest that prenylated flavonoids can simultaneously target both protein-level adhesion mechanisms and metabolic-level matrix biosynthesis pathways to compromise bacterial biofilms.

The superior antibacterial potency of ST3 and ST4 against *S. agalactiae* (MIC₉₀ = 3.12 μg/mL for both, compared with 6.25 μg/mL for norfloxacin) merits particular attention. *S. agalactiae* is a primary agent of contagious mastitis, and the escalating resistance to conventional antibiotics has created an urgent need for effective alternatives (Lacroix et al., 2023; Bacanlı, 2024). This finding extends our understanding of the antimicrobial spectrum of these compounds. Our previous studies established their potent activity against phytopathogenic fungi such as *B. cinerea* (EC₅₀ = 16.55 μg/mL for kurarinone; An et al., 2024) and phytopathogenic bacteria such as *Xoo* (MIC₅₀ = 1.56 μg/mL for sophoraflavanone G; An et al., 2025). The present work now demonstrates that their antibacterial efficacy extends to veterinary pathogens of major economic importance, with MIC values against *S. agalactiae* surpassing those of a clinically used antibiotic. This cross-kingdom antimicrobial spectrum—from phytopathogens to animal pathogens—highlights the broad potential of these compounds. The consistent superiority of ST4 over ST3 across multiple assays (biofilm inhibition, qPCR, EPS reduction) also provides useful structure–activity relationship insights: the presence of a carbonyl group at the C3 position in ST4, which can form intramolecular hydrogen bonds with the C7 hydroxyl group, may enhance receptor binding and biological potency, a trend also observed in our structure–activity analysis of these compounds against phytopathogenic fungi (An et al., 2024).

The first arm of the antibiofilm action—binding affinity to adhesion-related proteins—represents a mode of action not previously associated with prenylated flavonoids. In the present study, these compounds exhibited strong binding affinity to FbsA, FbsB, FbsC, and Lmb, adhesion-related proteins that mediate critical host–pathogen interactions during *S. agalactiae* colonization of mammary epithelium (Zhang et al., 2019; Aratani, 2018; Xu W. et al., 2025), and transcriptionally downregulated their encoding genes. We note that molecular docking was performed selectively on ST3 and ST4 as the two most potent compounds, with the aim of exploring potential factors underlying their differential biological activity. This analysis was not intended to establish exclusive targeting of these proteins or to construct a comprehensive structure–activity relationship across all 15 compounds. The correlation between docking scores and biological potency, while suggestive, does not establish a causal link between adhesion protein binding and antibacterial activity. The molecular docking results presented here are computational predictions and do not constitute direct experimental evidence of protein–ligand binding. Orthogonal biophysical validation using techniques such as surface plasmon resonance (SPR), isothermal titration calorimetry (ITC), or microscale thermophoresis (MST) will be necessary to confirm these interactions in future studies. We also acknowledge that pLDDT scores from AlphaFold were not systematically recorded during the initial modeling for FbsA, FbsB, and FbsC; future computational studies would benefit from the inclusion of these and other comprehensive model quality metrics to further validate the predicted structures. Our earlier work demonstrated that sophoraflavanone G (ST4) inhibited biofilm formation in *Xoo* primarily by suppressing chemotaxis and flagellar motility, with molecular docking showing binding to chemotaxis-related proteins (CheW, FliA, CheY, CheZ, FliM; An et al., 2025). In *B. cinerea*, kurarinone and sophoraflavanone G were shown to disrupt cell wall integrity via chitinase and *β*-1,3-glucanase activation and to damage cell membranes through ergosterol reduction and ROS-induced lipid peroxidation (An et al., 2024). The strong correlation between docking scores and biological potency (ST4 > ST3 for FbsC binding, biofilm inhibition, and gene downregulation) is consistent with the hypothesis that this interaction may contribute to the differential antibiofilm activity observed between these two compounds. A systematic docking analysis of the full compound library, combined with orthogonal biophysical validation, will be necessary to confirm these relationships. These findings suggest that prenylated flavonoids can suppress specific bacterial adhesion proteins at the transcriptional level to disrupt biofilm formation—a mechanism distinct from the membrane-disruptive effects observed in *S. aureus* and *B. cinerea* (Weng et al., 2024; An et al., 2024; Miladi et al., 2016), or the chemotaxis-inhibitory effects observed in *Xoo* (An et al., 2025).

The second arm of the mechanism—perturbation of the ABC transporter pathway—was suggested by untargeted metabolomics and provides new insight into how prenylated flavonoids interfere with biofilm matrix biosynthesis. In our previous study on *Xoo*, transcriptomic analysis revealed that sophoraflavanone G affected the two-component system, ribosome, bacterial chemotaxis, flagellar assembly, and oxidative phosphorylation pathways, with EPS reduction being attributed to transcriptional downregulation of *gum* genes *(gumB, gumD, gumG*; An et al., 2025). The involvement of the ABC transporter pathway was not identified in that study, which employed transcriptomic rather than metabolomic profiling and instead revealed the two-component system, chemotaxis, flagellar assembly, and oxidative phosphorylation as the primary affected pathways (An et al., 2025). This methodological complementarity underscores the value of multi-omics approaches and suggests that the ABC transporter pathway represents a metabolically distinct target. In the present work, KEGG enrichment analysis of untargeted metabolomic data identified the ABC transporter pathway as significantly affected by both ST3 and ST4 in *S. agalactiae.* The classification of differential metabolites provided additional context for this finding. In both treatment groups, flavonoids accounted for a substantial proportion of the differential metabolites (17.14% in the ST3 group), and several of the enriched metabolites—including amicoumacin B and 8-prenylnaringenin—have documented antibacterial or antibiofilm activities. The enrichment of flavonoid-related metabolites suggests that the administered compounds and their structural analogs may participate in the antibiofilm mechanism. However, we acknowledge that these metabolomic observations are correlative, and further targeted studies are needed to establish the functional roles of individual metabolites. Direct measurement of ABC transporter activity—including ATPase activity assays. Western blot analysis of transporter protein expression, or substrate transport measurements—was not performed in this study and will be necessary to establish a definitive causal link between pathway perturbation and reduced EPS secretion. The functional relationship between ABC transporter perturbation and the observed changes in biofilm matrix components warrants further elaboration. ABC transporters mediate the ATP-dependent translocation of substrates across the cell membrane, and specific ABC transporter systems have been implicated in the export of extracellular polysaccharides that constitute the structural scaffold of the biofilm matrix (Ghate et al., 2021; Wang et al., 2020). The significant reduction in both water-soluble and water-insoluble EPS observed in our study (by up to 60%) is consistent with impaired polysaccharide secretion through this pathway. The near-complete loss of capsular polysaccharides visualized by TEM further supports the interpretation that the polysaccharide export machinery has been functionally compromised by the compounds. The reduction in eDNA—another critical biofilm matrix component—may be a secondary consequence of biofilm matrix destabilization, as the eDNA scaffold is structurally integrated with polysaccharides and proteins within the biofilm architecture, and the disruption of one matrix component can lead to the disassembly of others.

We acknowledge that changes in membrane permeability could, in principle, produce similar effects on extracellular matrix secretion. However, several lines of evidence in our study argue against this as the primary mechanism. First, the KEGG enrichment analysis specifically identified the ABC transporter pathway as significantly affected, rather than pathways associated with membrane damage or cell wall stress responses. Second, the antibiofilm effects were observed at sub-MIC concentrations (0.39–1.56 μg/mL), where we did not observe significant bactericidal activity—an outcome that would be expected if membrane disruption were the primary mode of action. Third, previous studies have reported that ST3 and ST4 can cause membrane disruption in *S. aureus* and *B. cinerea*, but those effects were associated with substantial killing at comparable concentrations, whereas our time-killing curves showed minimal effects on viability at the concentrations tested in our biofilm assays. While we cannot completely exclude a contributory role of membrane effects, the collective evidence suggests that ABC transporter perturbation is the more plausible primary mechanism underlying the reduced secretion of biofilm matrix components at the sub-MIC concentrations tested. Consistent with this interpretation, our phenotypic validation experiments confirmed that this pathway perturbation has tangible consequences: EPS production was reduced by up to 60%, capsular polysaccharides were nearly completely stripped from the bacterial surface (as visualized by TEM), and eDNA release was significantly diminished by approximately 40%. To the best of our knowledge, this is the first study to report an association between prenylated flavonoid treatment and ABC transporter-mediated suppression of biofilm matrix secretion in bacteria. The discovery that these compounds affect EPS secretion through a different pathway in *S. agalactiae* (ABC transporter) than the one identified in *Xoo* (*gum* gene downregulation) suggests that prenylated flavonoids may engage species-specific or pathway-specific targets depending on the bacterial context, a finding that warrants further comparative investigation.

While ST4 demonstrated superior overall antibiofilm activity across the majority of endpoints assessed—including biofilm biomass, EPS production, and adhesion gene downregulation—we note that this superiority was not uniform across all matrix components or concentrations. At the lower concentration tested (0.78 μg/mL), ST3 inhibited eDNA release more potently than ST4 (36.2% vs. 10.1%), whereas at 1.56 μg/mL, both compounds reduced eDNA to a comparable extent (~40%). These nuanced differences indicate that the relative activities of these two structurally similar compounds may vary depending on the specific matrix component and concentration examined, and highlight the complexity of biofilm matrix assembly.

The anti-virulence and antibiofilm strategies demonstrated by prenylated flavonoids from *S. flavescens* in this study are part of a growing body of research on natural products from traditional medicinal plants. For instance, carvone, a monoterpene from aromatic plants, has been reported to inhibit virulence factor production, reduce biofilm formation, and enhance antibiotic susceptibility in *Serratia marcescens* (Zhou et al., 2024). Similarly, quorum sensing inhibitors have been extensively explored as strategies to attenuate the virulence and drug resistance of *Pseudomonas aeruginosa* (Zhou et al., 2025). Notably, prenylated flavonoids from other plant sources have also demonstrated dual-action antimicrobial mechanisms. Li et al. (in press) recently reported that prenylated flavonoids from paper mulberry (*Broussonetia papyrifera*) fruit exhibited potent anti-staphylococcal activity through a dual mechanism involving disruption of cell structure and inhibition of fatty acid biosynthesis by targeting PlsC, as supported by molecular docking studies. This finding is particularly relevant to our study, as it illustrates that prenylated flavonoids from phylogenetically unrelated plant species can exert antibacterial effects through multiple complementary targets, a concept echoed in our observation that kurarinone and sophoraflavanone G simultaneously target adhesion-related proteins and the ABC transporter pathway. These diverse examples, together with our findings, underscore the potential of plant-derived prenylated flavonoids as promising sources of multi-target antimicrobial agents.

The coordinated action of these two pathways—one preventing bacterial adhesion to host tissues, the other compromising the biofilm matrix that protects embedded bacteria—likely accounts for the robust antibiofilm effects observed at sub-MIC concentrations (0.39–1.56 μg/mL). This is a therapeutically important feature: even at doses insufficient to completely kill bacteria, these compounds can effectively disarm them and prevent biofilm establishment or persistence. This sub-MIC antibiofilm activity was not explicitly demonstrated in our earlier studies on phytopathogens (An et al., 2024; An et al., 2025) and represents an additional advantage for potential clinical or veterinary applications, where complete sterilization may not always be achievable. Furthermore, the dual-pathway nature of the antibiofilm activity—targeting two independent processes (adhesion protein expression and metabolic matrix biosynthesis)—may reduce the likelihood of resistance development, as bacteria would need to simultaneously acquire mutations in multiple unrelated targets to evade both effects (Chen et al., 2022; Boozari et al., 2019).

The *in vivo* efficacy of ST3 and ST4 was validated in a mouse mastitis model. While our previous studies on these compounds were limited to *in vitro* characterization (An et al., 2024; An et al., 2025), the present work provides, to our knowledge, the first *in vivo* evaluation of kurarinone and sophoraflavanone G for mastitis control. Intragastric administration significantly ameliorated histopathological lesions in mammary tissue, reduced the expression of pro-inflammatory cytokines (TNF-*α*, IL-1β, IL-6, IL-8), and decreased MPO activity—a marker of neutrophil infiltration—by 33.68 and 22.96% for ST3 and ST4, respectively. These *in vivo* anti-inflammatory effects are consistent with the well-documented anti-inflammatory properties of prenylated flavonoids reported in other models (Weng et al., 2024) and are likely attributable to a combination of reduced bacterial burden (due to antibacterial and antibiofilm activities) and direct modulation of host inflammatory signaling. The dual antibacterial and anti-inflammatory action is particularly desirable for mastitis therapy, as excessive inflammation is a major contributor to mammary tissue damage and impaired milk production (Soteriades et al., 2019; Martí-De Olives et al., 2020). This dual action—targeting both the pathogen and the host inflammatory response—distinguishes these compounds from most conventional antibiotics, which typically lack intrinsic anti-inflammatory properties.

It is noteworthy that the relative potencies of ST3 and ST4 differed between the *in vitro* and *in vivo* assays. *In vitro*, ST4 consistently outperformed ST3 across biofilm inhibition, *FbsB* downregulation, and EPS reduction. However, in the mouse mastitis model, ST3 showed greater reductions in IL-1β (44.8% vs. 12.3%), TNF-α (35.9% vs. 28.5%), and MPO activity (33.7% vs. 23.0%). This apparent reversal may reflect differences in the pharmacokinetic properties of the two compounds—including absorption, distribution, metabolism, and mammary gland penetration—that influence target tissue exposure independently of intrinsic antibacterial activity. It is well recognized that *in vitro* potency does not always predict *in vivo* efficacy, given the complex pharmacokinetic factors involved. Importantly, both ST3 and ST4 demonstrated significant therapeutic effects in the mastitis model, supporting their potential for further development regardless of their relative ranking in specific endpoints. Future pharmacokinetic studies comparing these two closely related flavonoids will be necessary to clarify the basis for their differential *in vivo* efficacy.

Safety assessment confirmed favorable toxicological profiles for both compounds. At concentrations effective against *S. agalactiae* (1.56–3.12 μg/mL), minimal or no cytotoxicity was observed against RAW264.7 macrophages and HIEC intestinal epithelial cells. The acute intragastric LD₅₀ > 2000 mg/kg in mice classifies both compounds as low-toxicity agents according to OECD guidelines (OECD, 2002). These findings are consistent with the traditional use of *S. flavescens* in herbal medicine and with previous safety evaluations of prenylated flavonoids (Lv et al., 2023; Weng et al., 2024). The favorable selectivity between antibacterial efficacy and mammalian cell cytotoxicity is particularly encouraging for the development of these compounds as feed additives or therapeutic agents for dairy cattle.

This study has several limitations that should be acknowledged and addressed in future work. First, the mouse mastitis model, while widely used as a proof-of-concept system, does not fully replicate the anatomy, physiology, and pharmacokinetics of the bovine mammary gland. Additionally, the intragastric route of administration used in this study results in systemic drug exposure, whereas the clinical treatment of bovine mastitis typically involves intramammary infusion for targeted delivery to the site of infection. The pharmacokinetic profile and mammary gland penetration of these compounds following different routes of administration remain to be investigated. Efficacy studies in dairy cattle, preferably with naturally occurring mastitis, will be essential to confirm translational potential. Second, the compounds were administered intragastrically in the present study; for practical application as feed additives, long-term oral administration studies in cattle evaluating stability, bioavailability, optimal dosing formulations, and effects on rumen microbiota are required. Furthermore, the dose of 150 mg/kg used in mice, while showing efficacy and safety (LD₅₀ > 2000 mg/kg), should not be directly extrapolated to cattle due to substantial differences in metabolic rate, body mass, and drug disposition between rodents and ruminants. The favorable safety profile provides a wide therapeutic window, suggesting that effective concentrations may be achievable in target tissues; however, dedicated pharmacokinetic studies in cattle, including assessment of mammary gland drug concentrations following both systemic and intramammary administration, will be necessary to determine clinically relevant dosing regimens. Nevertheless, the favorable safety profile (LD₅₀ > 2000 mg/kg) and the dual-pathway antibiofilm activity demonstrated here—particularly the suppression of adhesion proteins critical for mammary epithelial colonization—provide a rational basis for designing future clinical trials in dairy cattle with naturally occurring mastitis. Third, while we have established the involvement of the ABC transporter pathway through metabolomics and phenotypic validation, the precise molecular targets within this pathway—i.e., which specific ABC transporters are directly or indirectly affected by ST3 and ST4—remain to be identified through techniques such as gene knockout, proteomic pull-down assays, or surface plasmon resonance. Fourth, residue depletion studies in milk from treated animals will be necessary to establish appropriate withdrawal periods and ensure food safety. Finally, the comparative finding that prenylated flavonoids engage different pathways in different bacterial species (ABC transporter in *S. agalactiae* vs. *gum* gene regulation and chemotaxis in *Xoo*) merits systematic investigation to determine whether these represent truly species-specific mechanisms or context-dependent manifestations of a common upstream target.

Despite these limitations, the present findings establish a solid scientific foundation for further development. The combination of superior *in vitro* antibacterial activity, a dual antibiofilm action involving adhesion protein suppression and ABC transporter pathway perturbation, *in vivo* efficacy in a validated animal model, and favorable safety profiles distinguishes this work not only from previous reports on prenylated flavonoids by other groups, but also extends our own prior research (An et al., 2024; An et al., 2025) in significant new directions—from phytopathogens to veterinary pathogens, from single-mechanism studies to integrated multi-target elucidation, and from *in vitro* characterization to *in vivo* proof-of-concept. The present study thus positions kurarinone and sophoraflavanone G as promising candidates for further development as sustainable antibiotic alternatives for bovine mastitis control. Network pharmacology approaches may offer a valuable complementary strategy for future studies to explore the multi-target mechanisms of these compounds and to identify potential additional molecular targets beyond the adhesion proteins and ABC transporter pathway characterized here.

## 5 Conclusion

This study demonstrates that kurarinone and sophoraflavanone G, two prenylated flavonoids isolated from *S. flavescens*, exhibit potent antibacterial activity against *S. agalactiae*, with MIC₉₀ values of 3.12 μg/mL against *S. agalactiae* superior to that of the positive control norfloxacin (6.25 μg/mL). The most significant contribution of this work is the demonstration of a dual antibiofilm action involving two complementary pathways. Mechanistically, these compounds exhibited strong binding affinity to the adhesion-related proteins FbsA, FbsB, FbsC, and Lmb in molecular docking analysis, with transcriptional downregulation of their encoding genes confirmed by qPCR, and perturbed the ABC transporter pathway, as suggested by untargeted metabolomics, resulting in markedly suppressed secretion of EPS, capsular polysaccharides, and extracellular DNA. This dual antibiofilm action—simultaneously preventing bacterial adhesion to host tissues and compromising the structural integrity of the biofilm matrix—operates at sub-MIC concentrations and represents a mode of action distinct from the previously reported membrane-disruptive or chemotaxis-inhibitory effects of these compounds. In a mouse mastitis model, both compounds significantly ameliorated mammary tissue histopathological lesions, reduced the expression of pro-inflammatory cytokines (TNF-*α*, IL-1β, IL-6, IL-8), and decreased MPO activity, while exhibiting low cytotoxicity *in vitro* and an acute intragastric LD₅₀ > 2000 mg/kg *in vivo*. These findings position kurarinone and sophoraflavanone G as promising candidates for the control of biofilm-associated bovine mastitis, and provide a scientific basis for further development of *S. flavescens*-derived prenylated flavonoids as sustainable antibiotic alternatives for dairy production.

## Data Availability

The original contributions presented in the study are included in the article/Supplementary material, further inquiries can be directed to the corresponding authors.
