# Supplementary material for: Kurarinone and sophoraflavanone G from Sophora flavescens suppress Streptococcus agalactiae biofilm via dual antibiofilm mechanisms involving adhesion protein binding and ABC transporter pathway perturbation
Source: Front Microbiol. 2026 Aug 31;17:1905109. doi: 10.3389/fmicb.2026.1905109 (PMC13572244; doi:10.3389/fmicb.2026.1905109)
Supplement: Supplementary file 1 [file Data_Sheet_1.DOCX]

Supplementary Figures

# 1 Ramachandran analysis of the protein structures used for molecular docking (**Supplementary** Figure 1)

# **Supplementary Figure 1.** Ramachandran analysis of the protein structures used for molecular docking. (A) Lmb (PDB ID: 3HJT). (B) FbsA (AlphaFold prediction). (C) FbsB (AlphaFold prediction). (D) FbsC (AlphaFold prediction). The percentages of residues in favored regions were >90% for Lmb, FbsA, and FbsC, and 87% for FbsB.

# 2 **¹H, ¹³C NMR spectra of the newly isolated compounds (ST5**–**ST8, ST11**–**ST15) and the lead compounds (ST3 and ST4))** (**Supplementary** Figures 2-23)

**Supplementary Figure 2.** The ^1^H NMR spectrum of ST3.

**Supplementary Figure 3.** The ^13^C NMR spectrum of ST3.

**Supplementary Figure 4.** The ^1^H NMR spectrum of ST4.

**Supplementary Figure 5.** The ^13^C NMR spectrum of ST4.

**Supplementary Figure 6.** The ^1^H NMR spectrum of ST5.

**Supplementary Figure 7.** The ^13^C NMR spectrum of ST5.

**Supplementary Figure 8.** The ^1^H NMR spectrum of ST6.

**Supplementary Figure 9.** The ^13^C NMR spectrum of ST6.

**Supplementary Figure 10.** The ^1^H NMR spectrum of ST7.

**Supplementary Figure 11.** The ^13^C NMR spectrum of ST7.

**Supplementary Figure 12.** The ^1^H NMR spectrum of ST8.

**Supplementary Figure 13.** The ^13^C NMR spectrum of ST8.

**Supplementary Figure 14.** The ^1^H NMR spectrum of ST11.

**Supplementary Figure 15.** The ^13^C NMR spectrum of ST11.

**Supplementary Figure 16.** The ^1^H NMR spectrum of ST12.

**Supplementary Figure 17.** The ^13^C NMR spectrum of ST12.

**Supplementary Figure 18.** The ^1^H NMR spectrum of ST13.

**Supplementary Figure 19.** The ^13^C NMR spectrum of ST13.

**Supplementary Figure 20.** The ^1^H NMR spectrum of ST14.

**Supplementary Figure 21.** The ^13^C NMR spectrum of ST14.

**Supplementary Figure 22.** The ^1^H NMR spectrum of ST15.

**Supplementary Figure 23.** The ^13^C NMR spectrum of ST15.

# 3 **Molecular docking of ST3 and ST4 with adhesion-related proteins FbsA, FbsB, FbsC, and Lmb** (**Supplementary** Figure 24)

**Supplementary Figure 24.** Molecular docking of ST3 and ST4 with adhesion-related proteins FbsA, FbsB, FbsC, and Lmb. (A) ST3 (kurarinone) docked with FbsA protein. (B) ST4 (sophoraflavanone G) docked with FbsA protein. (C) ST3 docked with FbsB protein. (D) ST4 docked with FbsB protein. (E) ST3 docked with FbsC protein. (F) ST4 docked with FbsC protein. (G) ST3 docked with Lmb protein. (H) ST4 docked with Lmb protein.
