# Supplementary material for: Kurarinone and sophoraflavanone G from Sophora flavescens suppress Streptococcus agalactiae biofilm via dual antibiofilm mechanisms involving adhesion protein binding and ABC transporter pathway perturbation
Source: Front Microbiol. 2026 Aug 31;17:1905109. doi: 10.3389/fmicb.2026.1905109 (PMC13572244; doi:10.3389/fmicb.2026.1905109)
Supplement: Supplementary file 2 [file Data_Sheet_2.DOCX]

Supplementary Data Sheet

# 1 Characterizations of the newly isolated compounds (ST5–ST8, ST11–ST15) and the lead compounds (ST3 and ST4).

**Data for compound ST3.** Yellow solid; ^1^H NMR (500 MHz, DMSO-*d6*) δ 7.22 (dd, *J* = 8.4, 2.1 Hz, 1H), 6.34 (d, *J* = 2.6 Hz, 1H), 6.26 (dd, *J* = 8.4, 2.3 Hz, 1H), 6.12 (s, 1H), 5.40 (ddd, *J* = 17.9, 13.2, 2.7 Hz, 1H), 4.92 (dt, *J* = 22.4, 7.0 Hz, 1H), 4.55 (t, *J* = 2.0 Hz, 1H), 4.48 (d, *J* = 2.6 Hz, 1H), 3.70 (s, 3H), 3.36 (s, 2H), 2.08 (s, 2H), 1.59 – 1.47 (m, 7H), 1.43 (s, 2H), 1.27 (d, *J* = 4.6 Hz, 1H), 0.86 (q, *J* = 7.2 Hz, 2H). ^13^C NMR (125 MHz, DMSO-*d6*) δ 189.40, 167.45, 162.91, 160.04, 158.43, 155.55, 148.35, 132.05, 131.07, 129.12, 127.74, 123.85, 111.16, 106.67, 104.80, 102.59, 92.81, 73.95, 55.71, 46.79, 44.75, 31.14, 25.94, 22.85, 19.04, 18.02. MS-ESI *m/z:* calcd for C26H30O6 : 439.2042[M+H]^+^ ; found: 439.2145.

**Data for compound ST4.** Pale yellow solid; ^1^H NMR (500 MHz, DMSO-*d*_6_) δ 7.22 (d, *J* = 9.0 Hz, 1H), 6.34 (d, *J* = 2.2 Hz, 1H), 6.27 (dt, *J* = 8.3, 1.8 Hz, 1H), 5.88 (s, 1H), 5.46 (dddd, *J* = 28.0, 13.3, 8.7, 4.8 Hz, 1H), 4.94 (ddt, *J* = 20.9, 14.2, 6.9 Hz, 1H), 4.57 (dt, *J* = 11.5, 2.1 Hz, 1H), 4.50 (dd, *J* = 12.9, 2.5 Hz, 1H), 3.16 (s, 2H), 1.85 (s, 1H), 1.65 (d, *J* = 2.4 Hz, 1H), 1.60 (s, 1H), 1.58 (s, 1H), 1.56 (s, 2H), 1.53 (s, 1H), 1.51 (s, 1H), 1.50 (s, 3H), 1.49 (s, 2H), 1.44 (s, 1H). ^13^C NMR (126 MHz, DMSO-*d*_6_) δ 196.30, 160.57, 158.17, 155.40, 155.28, 147.99, 147.66, 130.59 (d, *J* = 3.4 Hz), 127.53, 123.47, 116.32, 116.11, 110.69, 106.81, 106.68, 106.28 (d, *J* = 8.3 Hz), 102.34 (d, *J* = 9.8 Hz), 95.43, 48.56, 46.59 (d, *J* = 55.1 Hz), 31.03, 26.72, 25.56, 25.52, 18.65. MS-ESI *m/z*: calcd for C_25_H_28_O_6_: 425.1886[M+H]+; found: 425.1978.

**Data for compound ST5.** Pale yellow solid; ^1^H NMR (400 MHz, CDCl_3_) δ 7.60 (d, *J* = 8.7 Hz, 1H), 7.37 (d, *J* = 8.2 Hz, 1H), 6.46 (d, *J* = 3.1 Hz, 1H), 6.45 (s, 1H), 6.40 (d, *J* = 2.2 Hz, 1H), 5.61 (dd, *J* = 11.9, 4.5 Hz, 1H), 4.97 (d, *J* = 9.7 Hz, 1H), 4.54 (dt, *J* = 25.0, 1.9 Hz, 2H), 3.72 (d, *J* = 4.7 Hz, 3H), 2.84 – 2.72 (m, 2H), 2.65 (d, *J* = 7.3 Hz, 2H), 2.52 – 2.36 (m, 1H), 2.00 (m, *J* = 8.8 Hz, 2H), 1.61 (s, 3H), 1.56 (d, *J* = 7.9 Hz, 3H), 1.44 (s, 3H). ^13^C NMR (100 MHz, CDCl_3_) δ 193.87, 162.59, 162.51, 157.93, 157.26, 148.57, 131.65, 127.33, 126.02, 123.44, 119.14, 115.60, 114.08, 110.71, 109.76, 107.00, 98.71, 74.57, 55.22, 46.64, 43.30, 31.21, 27.53, 25.64, 18.95, 17.73. MS-ESI *m/z:* calcd for C_26_H_30_O_5_: 422.2093[M+H]^+^; found: 423.3204.

**Data for compound ST6.** Pale yellow solid; ^1^H NMR (400 MHz, CDCl3) δ 7.19 (d, *J* = 8.5 Hz, 2H), 6.79 – 6.75 (m, 2H), 5.87 (s, 1H), 5.16 (m, 2H), 3.28 (p, *J* = 1.7 Hz, 2H), 2.98 (dd, *J* = 17.2, 13.2 Hz, 1H), 2.64 – 2.58 (m, 1H), 1.68 (s, 3H), 1.59 (s, 3H). ^13^C NMR (100 MHz, CDCl_3_) δ 196.08, 174.16, 164.50, 157.43, 143.62, 131.83, 122.33, 115.50, 108.70, 102.26, 94.69, 79.07, 43.15, 25.67, 20.98, 17.64.. MS-ESI m/z: calcd for C_20_H_20_O_5_: 340.1311[M+H]^+^; found: 341.2502.

**Data for compound ST7.** Pale yellow solid; ^1^H NMR (500 MHz, DMSO-*d*_6_) δ 7.98 (d, *J* = 8.4 Hz, 2H), 6.92 (d, *J* = 8.3 Hz, 2H), 6.45 (s, 1H), 5.17 (s, 1H), 3.80 (s, 3H), 1.75 (s, 3H), 1.62 (s, 3H). ^13^C NMR (125 MHz, DMSO-*d*_6_) δ 171.31, 159.54, 158.53, 158.05, 155.49, 141.93, 131.07, 128.73, 122.77, 122.38, 115.47, 106.89, 105.30, 95.43, 55.81, 25.51, 21.55, 17.93. MS-ESI m/z: calcd for C_21_H_20_O_6_: 368.1260[M+H]^+^; found: 369.2406.

**Data for compound ST8.** Pale yellow solid; ^1^H NMR (400 MHz, CDCl_3_) *δ* 8.05 (2H, d, *J* = 8.9 Hz), 6.89 (2H, d, *J* = 8.9 Hz), 6.23 (1H, s), 5.19 (1H, td, *J* = 5.6, 2.3 Hz), 3.47 (2H, d, *J* = 6.8 Hz), 1.75 (3H, d, *J* = 1.6 Hz), 1.63 (3H, d, *J* = 1.8 Hz). ^13^C NMR (100 MHz, CDCl_3_) *δ* 175.7, 161.8, 158.8, 154.3, 146.5, 135.2, 132.3, 129.7, 122.8, 122.3, 115.6, 106.8, 103.3, 98.2, 25.7, 21.7, 17.9. MS-ESI m/z: calcd for C_20_H_18_O_6_: 354.1103[M+H]^+^; found: 355.2406.

**Data for compound ST11.** Yellow solid; ^1^H NMR (400 MHz, DMSO-d6) *δ* 14.85 (s, 1H), 7.88 (d, *J* = 5.8 Hz, 2H), 7.50 (d, *J* = 8.4 Hz, 1H), 6.52 – 6.44 (m, 2H), 6.08 (s, 1H), 5.75 (s, 1H), 4.97 (d, *J* = 1.8 Hz, 1H), 4.60 – 4.49 (m, 2H), 3.84 (d, *J* = 1.7 Hz, 6H), 2.48 (s, 1H), 2.08 (s, 1H), 2.02 (d, *J* = 7.3 Hz, 2H), 1.93 (d, *J* = 47.4 Hz, 1H), 1.65 (s, 3H), 1.59 (d, *J* = 1.6 Hz, 3H), 1.50 (d, *J* = 1.4 Hz, 3H). ^13^C NMR (100 MHz, DMSO-*d6*) δ 191.64, 165.31, 161.73, 160.40, 160.14, 147.91, 137.72, 130.63, 130.44, 123.77, 123.50, 114.77, 110.78, 108.45, 106.64, 104.23, 99.21, 90.94, 55.50, 55.47, 54.92, 46.15, 30.82, 25.59, 18.53, 17.68. MS-ESI *m/z:* calcd for C27H32O6 : 452.2199[M+H]^+^ ; found: 452.2288.

**Data for compound ST12.** Yellow solid; ^1^H NMR (400 MHz, DMSO-*d6*) δ 14.87 (s, 1H), 7.90 (d, *J* = 21.5 Hz, 1H), 7.43 (d, *J* = 8.6 Hz, 1H), 6.40 – 6.28 (m, 2H), 6.04 (s, 1H), 4.98 (m, *J* = 6.9, 3.2 Hz, 1H), 4.58 – 4.48 (m, 2H), 3.84 (s, 3H), 3.35 (s, 7H), 2.00 (d, *J*= 10.5 Hz, 3H), 1.65 (s, 3H), 1.61 – 1.58 (m, 3H), 1.52 – 1.49 (m, 3H). ^13^C NMR (100 MHz, DMSO-*d6*) δ 192.51, 165.75, 163.09, 161.62, 160.82, 159.49, 148.32, 139.17, 131.98, 130.94, 129.13, 123.92, 123.27, 114.23, 111.28, 108.54, 107.09, 104.94, 91.11, 65.49, 56.00, 46.64, 26.05, 18.97, 18.14. MS-ESI *m/z:* calcd for C26H30O6 : 438.2042[M+H]^+^ ; found: 439.2115.

**Data for compound ST13.** Pale yellow solid; ^1^H NMR (400 MHz, DMSO-*d*_6_) δ 7.75 (s, 1H), 7.61 (s, 1H), 7.51 (d, *J* = 6.9 Hz, 2H), 6.83 (d, *J* = 1.8 Hz, 2H), 6.02 (s, 1H), 5.16 (s, 1H), 3.79 (s, 3H), 3.10 (d, *J* = 7.1 Hz, 2H), 1.70 (d, *J* = 1.3 Hz, 3H), 1.59 (d, *J* = 1.5 Hz, 3H). ^13^C NMR (100 MHz, DMSO-*d_6_*) δ 189.89, 165.24, 161.15, 160.74, 141.61, 130.60, 129.44, 126.41, 124.48 (d, *J* = 5.2 Hz), 116.55, 108.03, 103.56, 93.01, 55.85, 55.39, 25.99, 21.72, 18.19. MS-ESI *m/z:* calcd for C_21_H_22_O_5_: 354.1467[M+H]^+^; found: 355.2603.

**Data for compound ST14.** Yellow solid; ^1^H NMR (400 MHz, CDCl_3_) δ 7.68 (s, 1H), 7.66 (s, 1H), 7.42 (d, *J* = 8.7 Hz, 2H), 6.80 – 6.77 (m, 2H), 5.91 (s, 1H), 5.22 – 5.15 (m, 1H), 3.82 (s, 3H), 3.26 (s, 2H), 1.73 (d, *J* = 1.5 Hz, 3H), 1.63 (d, *J* = 1.6 Hz, 3H).^13^C NMR (100 MHz, CDCl_3_) δ 192.90, 164.75, 161.84, 160.94, 159.05, 142.25, 132.44, 130.25, 127.43, 124.96, 122.60, 115.84, 107.96, 105.85, 90.84, 55.60, 25.76, 21.47, 17.78. MS-ESI *m/z:* calcd for C_21_H_22_O_5_: 354.1467[M+H]^+^; found: 355.2603.

**Data for compound ST15.** Pale yellow solid; ^1^H NMR (400 MHz, DMSO-*d*_6_) δ 7.71 (s, 2H), 7.59 (d, *J* = 8.7 Hz, 2H), 6.84 (d, *J* = 8.6 Hz, 2H), 6.54 (d, *J* = 10.0 Hz, 1H), 6.10 (s, 1H), 5.60 (d, *J* = 10.0 Hz, 1H), 3.92 (s, 3H), 1.41 (s, 6H). ^13^C NMR (100 MHz, DMSO-*d*_6_) δ 192.09, 162.38, 161.22, 160.32, 159.67, 143.50, 130.74, 126.00, 125.79, 123.28, 116.05, 115.25, 105.43, 101.99, 91.90, 78.07, 65.02, 56.35, 28.01. MS-ESI *m/z:* calcd for C_21_H_20_O_5_: 352.1311[M+H]^+^; found: 353.2303.
