# Supplementary material for: Kurarinone and sophoraflavanone G from Sophora flavescens suppress Streptococcus agalactiae biofilm via dual antibiofilm mechanisms involving adhesion protein binding and ABC transporter pathway perturbation
Source: Front Microbiol. 2026 Aug 31;17:1905109. doi: 10.3389/fmicb.2026.1905109 (PMC13572244; doi:10.3389/fmicb.2026.1905109)
Supplement: Supplementary file 3 [file Table_1.DOCX]

Supplementary Tables

**TABLE 1 Primer sequences used for qPCR amplification of *FbsA, FbsB, FbsC,* and *Lmb.***

| **Gene ID** | **Forward primer (5'~3')** | **Reverse primer (5'~3')** |
| --- | --- | --- |
| *FBSA* | AGCGTCGTCAACGTGATGCG | ACGCTCTAGAACATTGCCTTGGC |
| *FBSB* | GCGAGGTCATTTCCGCAGTT | ACTGCGCAAACTTCTGTCCA |
| *FBSC* | AGCTAAGCAGTCAAGACGTTCTC | TCAGTTGCGTGGCTTGTGTT |
| *LMB* | TGAATGCCTGCACCTGATTGGATC | ACGCAAGGCATGTCAGTTGTAACC |
| *16S* | GCTCACCAAGGCGACGATACATAG | GCGTTGCTCGGTCAGACTTCC |
| *TNF-α* | GGTGCCTATGTCTCAGCCTCTT | GCCATAGAACTGATGAGAGGGAG |
| IL-1β | TGGACCTTCCAGGATGAGGACA | GTTCATCTCGGAGCCTGTAGTG |
| *IL-6* | CTGCAAGAGACTTCCATCCAG | AGTGGTATAGACAGGTCTGTTGG |
| *IL-8* | TGTTCACAGGTGACTGCTCC | AGCCCATAGTGGAGTGGGAT |
| *β-actin* | GGCTGTATTCCCCTCCATCG | CCAGTTGGTAACAATGCCATGT |

**TABLE 2 Inhibition rates (%) of total flavonoids, compounds ST1–ST15, and norfloxacin at 100 μg/mL against four mastitis-associated bacterial species.**

| Test substance | Inhibition rate (%) | | | |
| --- | --- | --- | --- | --- |
|  | *S. agalactiae*^1^ | *S. dysgalactiae*^2^ | *E. coli*^3^ | *S. aureus*^4^ |
| Total flavonoids | 100.00±0.00^a^ | 100.00±0.00^a^ | 0.00±0.00^k^ | 22.47±2.67^h^ |
| ST1 | 99.20±0.77^a^ | 98.28±1.89^b^ | 20.9±1.85^f^ | 7.04±0.09^k^ |
| ST2 | 99.73±0.45^a^ | 98.97±0.27^b^ | 0.00±0.00^k^ | 99.41±0.30^a^ |
| ST3 | 100.00±0.00^a^ | 100.00±0.00^a^ | 0.00±0.00^k^ | 100.00±0.00^a^ |
| ST4 | 100.00±0.00^a^ | 100.00±0.00^a^ | 5.21±1.5^j^ | 100.00±0.00^a^ |
| ST5 | 83.30±2.02^e^ | 90.12±1.07^e^ | 4.47±0.79^j^ | 90.16±1.09^c^ |
| ST6 | 93.19±1.02^d^ | 95.03±1.13^c^ | 11.26±2.79^h^ | 85.36±1.07^d^ |
| ST7 | 39.43±2.01^g^ | 49.43±1.01^h^ | 29.34±4.11^d^ | 41.84±1.82^f^ |
| ST8 | 97.08±3.45^b^ | 61.17±2.12^f^ | 23.33±2.4^e^ | 23.44±1.18^h^ |
| ST9 | 39.38±1.45^g^ | 49.27±1.34^h^ | 42.7±1.03^c^ | 59.86±0.39^e^ |
| ST10 | 93.59±1.02^d^ | 100.00±0.00^a^ | 41.47±1.79^c^ | 100.00±0.00^a^ |
| ST11 | 5.44±1.47^j^ | 12.2±1.54^j^ | 14.7±2.32^g^ | 7.73±1.01^k^ |
| ST12 | 93.76±1.51^d^ | 95.95±2.57^c^ | 41.79±0.51^c^ | 95.24±2.71^b^ |
| ST13 | 95.73±0.67^c^ | 56.81±1.27^g^ | 7.81±1.04^i^ | 7.35±1.02^k^ |
| ST14 | 9.78±4.05^i^ | 17.26±2.09^i^ | 28.56±0.79^d^ | 33.44±1.45^g^ |
| ST15 | 5.62±1.84^j^ | 0.00±0.00^l^ | 0.00±0.00^k^ | 0.00±0.00^k^ |
| Norfloxacin | 100.00±0.00^a^ | 100.00±0.00^a^ | 100.00±0.00^a^ | 100.00±0.00^a^ |

Note: Data are presented as mean ± SD (*n* = 3), Different lowercase letters within the same row indicate significant differences among groups (*p* < 0.05, Duncan's multiple range test); ^1^*Streptococcus agalactiae* ATCC27956; ^2^*Streptococcus dysgalactiae* ATCC27957; ^3^*Escherichia coli* ATCC25922; ^4^*Staphylococcus aureus* Newman.
